# The Deep Correlation between Energy Metabolism and Reproduction: A View on the Effects of Nutrition for Women Fertility

**DOI:** 10.3390/nu8020087

**Published:** 2016-02-11

**Authors:** Roberta Fontana, Sara Della Torre

**Affiliations:** 1 Department of Pharmacological and Biomolecular Sciences, University of Milan, via Balzaretti 9, Milan 20133, Italy; roberta.fontana@iit.it; 2 Department of Drug Discovery and Development, Italian Institute of Technology, via Morego 30, Genova 16163, Italy; 3 Center of Excellence of Neurodegenerative Diseases, University of Milan, via Balzaretti 9, Milan 20133, Italy

**Keywords:** fertility, reproduction, women, energy metabolism, nutrients

## Abstract

In female mammals, mechanisms have been developed, throughout evolution, to integrate environmental, nutritional and hormonal cues in order to guarantee reproduction in favorable energetic conditions and to inhibit it in case of food scarcity. This metabolic strategy could be an advantage in nutritionally poor environments, but nowadays is affecting women’s health. The unlimited availability of nutrients, in association with reduced energy expenditure, leads to alterations in many metabolic pathways and to impairments in the finely tuned inter-relation between energy metabolism and reproduction, thereby affecting female fertility. Many energetic states could influence female reproductive health being under- and over-weight, obesity and strenuous physical activity are all conditions that alter the profiles of specific hormones, such as insulin and adipokines, thus impairing women fertility. Furthermore, specific classes of nutrients might affect female fertility by acting on particular signaling pathways. Dietary fatty acids, carbohydrates, proteins and food-associated components (such as endocrine disruptors) have *per se* physiological activities and their unbalanced intake, both in quantitative and qualitative terms, might impair metabolic homeostasis and fertility in premenopausal women. Even though we are far from identifying a “fertility diet”, lifestyle and dietary interventions might represent a promising and invaluable strategy to manage infertility in premenopausal women.

## 1. Introduction

Infertility, which is defined as not being able to conceive after one year of unprotected sex, is an ongoing problem estimated to affect 186 million people worldwide [1]; in some regions of the world, such as in developing countries, the percentage of infertility could reach an average of 30% [2]. Although male infertility contributes to more than half of all cases of global childlessness, infertility remains a woman’s social burden [1]. The etiologies of female infertility include ovulation and tubal problems, endometriosis and in 20%–30% cases remain unexplained [3]. Recently, the effects of lifestyle on female reproductive health have received new attention [3,4,5]. Body weight, body composition, physical activity, and nutrients intake are all factors that could impact female fertility [4].

In female animals, reproduction and metabolism are tightly connected and reciprocally regulated [6,7]. During the reproductive period of life, the physiological activity of the gonads, with their cyclic production of sex hormones, ensures continuous regulation of energy metabolism [6,7]. On the other hand, in females, in particular in mammals, energy metabolism is tuned on reproductive needs: the energetic costs of puberty, pregnancy and lactation rely on female ability in saving oxidizable fuels [6,8]. Throughout evolution mechanisms have been developed to store energy in the case of food abundance and to prevent reproduction in nutrient poor environments [9,10]. When this physiological balance between reproduction and metabolism is disrupted problems occur. Pathologies associated with ovarian dysfunction, such as polycystic ovarian syndrome (PCOS) or Turner syndrome are generally more susceptible to developing metabolic disturbances [11,12], and physiologically, after the cessation of ovarian function, women are also at higher risk for developing metabolic and cardiovascular disorders [13]. On the other hand, in our obesogenic world, the gender-specific strategy developed throughout evolution to guarantee the survival of the species has negative effects on female health [9] and is now also impacting women’s fertility [14].

Many studies reveal the J-shaped relation between body mass index (BMI) and infertility; indeed, the effect of body mass on fecundity appeared to be bimodal. Underweight (BMI < 19 kg/m^2^) and overweight (BMI 25–29.9 kg/m^2^) women have similar risk of infertility; on the other hand, morbidly obese women (BMI > 30 kg/m^2^) have more than two fold greater risk of ovulatory disorders [4,5]. The impact of BMI on reproduction seems to be specific for females pointing, once again, to a sex dimorphism in the mechanisms linking reproduction and metabolism. Indeed, being overweight impairs fertility in women more than it does in men [15].

Given the tight interconnection between energy metabolism and reproduction, in this review, we discuss the impact that the metabolic state, and related hormones (such as insulin and adipokines), could have on fertility in women considering underweightness and exercise but also overweightness and obesity. Moreover, we discuss the impact of different classes of macronutrients (fatty acids, carbohydrates, and proteins) and of some food-associated components (endocrine disruptors) on female health and fertility. Finally, we report the beneficial effects that Mediterranean diet was suggested to have on women’s reproductive health.

## 2. Estrogen Receptor, the Link between Energy Metabolism and Reproduction

There is undoubtedly a tight interconnection between energy metabolism and fertility, above all in females. This relation appears in less evolved animals, such as nematodes and insects: in these species, indeed, signaling coming from metabolic organs senses energy reserves and in case of food scarcity inhibits reproduction [6,16]. Recently, we reviewed the mechanisms that link female fertility and energy metabolism throughout evolution, revealing that a central role in this regulation has been gained by estrogens and their cognate receptors [6]. Estrogenic control over reproduction has long been known and extensively studied. In the last years, their role in the control of energy metabolism is also emerging [17,18]. Estrogen receptor alpha (ERα) is the one that mediates most of the estrogenic effects on energy homeostasis: knock out mice for ERα, which are infertile, have increased body weight and food intake when compared to their wild-type littermates [19].

In the central nervous system (CNS), particularly in the hypothalamus, which is the central regulator of energy homeostasis, estrogens were demonstrated to decrease food intake, increase energy expenditure and promote fat distribution to subcutaneous rather than visceral depots [20,21,22]. More in detail, estrogens regulate the expression of orexigenic neuropeptides, such as neuropeptide Y (NPY) and agouti-related protein (AgRP), and the activity of anorexigenic neurons, such as proopiomelanocortin (POMC) and cocaine- and amphetamine-regulated transcript (CART) [20,21,22]. Additionally, estrogens, acting both in the hypothalamus and in brainstem, potentiate the anorexigenic signaling and attenuate the orexigenic ones produced by various peptides coming from the periphery, such as cholecystokinin, leptin and ghrelin [23,24,25].

In the periphery, estrogens act at different levels. It is well documented that fertile-age women have a subcutaneous instead of a visceral distribution of fat mass [18]. This effect seems to be dependent upon estrogens activity: in post-menopausal women, when estrogenic signaling decreases, the subcutaneous fat redistributes to the visceral area [18,26]. Additionally, estrogens were demonstrated to have an anti-lipogenic and pro-lipolytic activity in adipocytes [27,28]. Estrogens also promote pancreatic β cells activity: they directly stimulate insulin biosynthesis and inhibit lipid accumulation thus preventing lipotoxicity and promoting β cells survival [29,30,31]. In the liver, ERα, the main isoform of the receptor in this tissue [32], has a relevant role in lipid homeostasis [33]. Studies conducted in our and other laboratories demonstrated that hepatic ERα signalling regulates the expression of many genes, the products of which are involved in fatty acid and cholesterol metabolism [33,34]. Additionally, we demonstrated that hepatic ERα is a sensor of the metabolic signals, and in particular of amino acids, able to tune energy metabolism on reproductive needs [10].

## 3. Underweight and Exercise and Reproductive Outcome

When energy is scarce the mechanisms that partition energy favor the processes that ensure the survival of the individuals over those promoting growth and reproduction [16]; this ancestral mechanism is also true in women. Hassan and Killick showed that underweight women (BMI < 19 kg/m^2^) have a four-fold longer time to pregnancy than women with a normal BMI. Specifically, underweight women required an average of 29 months to conceive as compared to 6.8 months in women with a normal weight profile [5,35]. Indeed, as for the initiation of menses, a minimum of fat mass is necessary, for maintaining ovulatory function [36]. Conditions of energy deficit, such as eating disorders (ED), malnutrition and strenuous physical activity, are associated with subfecundity and infertility [36,37,38].

Undernutrition due to low food availability is not common in developed countries but, in poorer ones, is certainly connected with infertility risk [36]. In developed nations, conditions that produce malnutrition are eating disorders (ED), a spectrum of psychiatric disturbances that affects childbearing age women [39]. EDs are associated with hypothalamic amenorrhea, oligomenorrhea, anovulatory cycles, and luteal phase deficiency [31,37]. At the level of the CNS, food deprivation was demonstrated to inhibit the hypothalamic-pituitary-gonads axis (HPG), affecting GnRH pulse generator. Inhibition of GnRH secretion leads to a cascade of inhibitory effects, including decreased gonadotropin secretion, retarded follicle development, and inhibited synthesis of gonadal steroids [16,40,41,42].

Nutritional signals coming from the periphery are also important for the regulation of reproduction. Studies conducted in our laboratory showed that hepatic ERα is an important mediator of these effects [10]. Calorie restriction induces a decrease in liver ERα activity, which is associated with a disruption of the estrous cycle, whereas dietary amino acids (AAs) prevent this effect by promoting hepatic ERα activity, inducing liver IGF-1 synthesis and enabling estrous cycle progression [10].

Physical activity (PA) is also associated with menarche and reproductive health [43]. Frequency, duration and intensity of PA are all correlated with subfecundity and infertility [38]. Precisely, moderate exercise and weight loss improve metabolic function and hormonal profile in obese women, often leading to increase fertility [44,45]. On the other hand, women that exercise to exhaustion have 2.3–3 fold increased risk of infertility [38]. While, for a long time, it was believed that ovarian disturbances in athletes could be dependent on fat mass, it is now appearing clear that physical activity effects on reproduction are independent of body fat stores [46]. Menstrual cycle returns in female athletes when energy expenditure is reduced even without changes in body weight and fat mass [46,47]. It is plausible that is the negative energy balance due to high loads of exercise not coupled with increased energy intake, which leads to menstrual cycle disruption [46,48].

## 4. Obesity and Fertility

The global obesity epidemic is now pandemic [49]. In 2009–2010, the prevalence of obesity in the United States was 35.5% among adult men and 35.8% among adult women [50]; in 2011–2012, 32.2% of children in the US aged 2 to 19 years were overweight and 17.3% were obese [51]. Obesity is associated with the development of type 2 diabetes mellitus, coronary heart disease, certain forms of cancer and multiple alteration of the endocrine system, such as PCOS [52,53,54].

**Figure 1:**
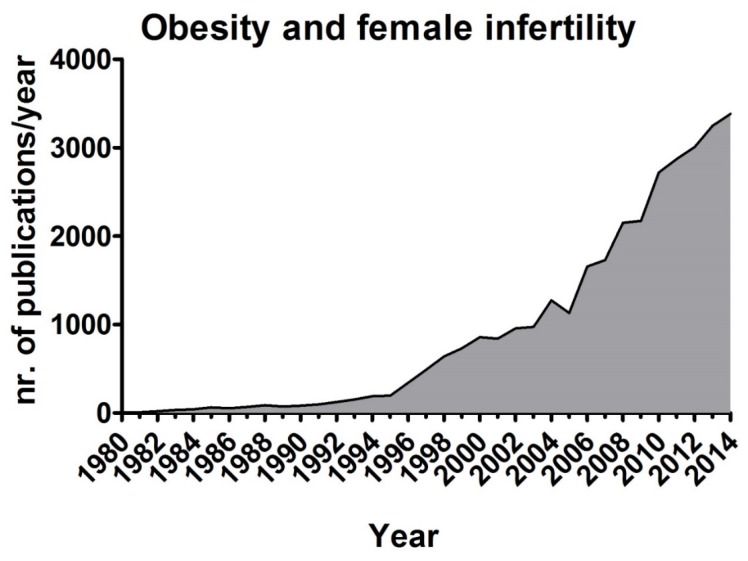
Number of publications reporting the association between female infertility and obesity. The great increase in number of reports appeared per year since 1980 reveals that the interest in the topic has increased during the last decades. Data were obtained using WOS (Web of Science).

Childhood BMI is correlated to pubertal development: greater adiposity was reported to induce menarche at earlier age [55]. This predisposes women to higher risk of obesity, diabetes and breast cancer [55,56,57]. Additionally, obesity clearly increases women’s risk of fertility impairment, in particular menstrual disorders, infertility, miscarriage, poor pregnancy outcomes, and impaired fetal well-being [14,58,59]. This topic is appealing and alarming, as demonstrated by the increased number of publications appeared in the last 35 years (Figure 1). In ovulatory but subfertile women the chance of spontaneous conception decreases by 5% for each unit increase in the BMI (body mass index) exceeding 29 kg/m^2^ [5,60]. Overweightness and obesity are also associated with negative outcomes for patients undergoing *in vitro* fertilization [14,61]. Indeed, the increased risk of subfecundity and infertility in overweight and obese women is associated with not only impairment at the level of the HPG axis, but also oocyte quality and uterine receptivity [61,62]. Given this, it is recommend overweight and obese women lose weight in order to improve fertility [5]. Overweightness effects on fertility seem to be sexually dimorphic. In a study conducted in 483 men, it was observed that BMI was unrelated to sperm concentration, motility and morphology [15]. Only in obese subjects, the total sperm count was lower in comparison to normal weight men [15].

Dyslipidemia is often observed in obese patients, with increased plasmatic triglycerides and free fatty acids, decreased HDL-C (high-density lipoprotein cholesterol) and slightly increased in LDL-C (low-density lipoprotein cholesterol) [63]. There is a body of literature pointing out the role of lipids for female fecundity; indeed, cholesterol and fatty acids are determinant for reproductive function at the level of ovary, uterus and placenta [64,65,66]. Recently, serum free cholesterol concentrations, in both women and men, have been associated with reduced fecundity [67]. An abnormal lipoprotein metabolism has been correlated to dysfunctional oocyte and infertility, as observed in SR-BI (scavenger receptor BI) gene knock out mice [68]. Using this model, the authors clearly showed the existence of a hepatic-ovarian axis necessary for female reproductive function. This mechanism has never been extensively studied although it could be a key in linking reproduction with metabolism. A recent work performed in our laboratory revealed that, in female mice, the estrous cycle coordinates cholesterol homeostasis and that hepatic ERα is necessary for this effect [69]. Altogether, these data further highlight that the influence of metabolism on reproduction is bidirectional.

Finally, insulin resistance is often correlated with obesity; the effect of this pancreatic hormone on female fertility has been extensively studied and is reviewed in the next section.

## 5. Insulin and Adipokines: The Most Involved Molecular Players Linking Obesity and Reproductive Impairment

**Figure 2:**
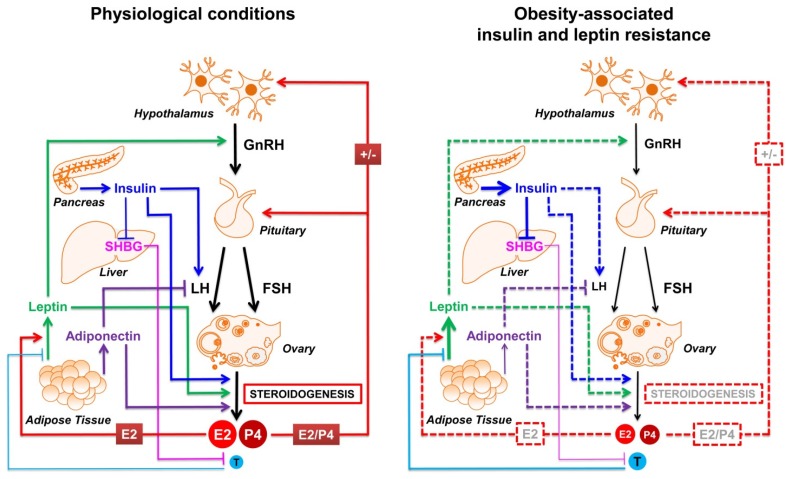
Effects of insulin, leptin and adiponectin on the regulation of hypothalamic pituitary gonadal axis (HPG) and steroidogenesis under physiological and obesity-associated conditions. Under physiological conditions (**left**) the signaling pathways of insulin, leptin and adiponectin are able to sense the nutritional status, and, jointly with sex hormones sustain ovarian activity and reproduction. In obesity (**right**), the increased levels of insulin and leptin lead to insulin and leptin resistance that, together with the reduced adiponectin levels, contribute to a dysregulation of both the HPG axis and ovarian steroidogenesis, by further worsening the endocrine milieu (impaired E2 and P4 synthesis, increased T synthesis and free levels due to reduced SHBG synthesis) and impairing ovarian function and fertility success rate. In the figure the dotted lines represent impaired regulations. E2 = 17β-estradiol; P4 = progesterone; T = testosterone.

From what is said above, it appears clear that energy metabolism and female fertility are tightly connected and reciprocally regulated. Not surprisingly many peripheral signals report nutritional status to the CNS and to the ovary in order to coordinate reproduction. Undoubtedly, hormones derived from the gut, adipose tissue and pancreas (such as peptide YY, adipokines, insulin and others) were recognized as regulating human reproduction, as these tissues are ideally placed to sense and detect nutritional intake and status [70,71]. In the sections below, we decided to focus our discussion on the signal of insulin and adipokines as they are largely described to mediate the negative impact that obesity has on fertility, highlighting their effects at the level of the HPG axis and their influence on steroidogenesis, as summarized in Figure 2.

### 5.1. Insulin

Insulin has long been known as a peripheral regulator of energy homeostasis: this hormone controls glucose uptake, oxidation and storage [72]. In particular, once secreted by pancreatic β cells in response to increased blood glucose levels, insulin stimulates glucose uptake by the skeletal muscle and by the adipose tissue and regulates lipid metabolism in the liver.

Insulin resistance (IR) is a disorder characterized by an impaired metabolic response to either exogenous or endogenous insulin, with consequences on carbohydrate, lipid and protein metabolism [73]. Pancreatic β cells try to counteract insulin resistance by enhancing their mass and secretory activity; however, when the functional expansion of islet β-cells fails to compensate, insulin deficiency and ultimately type 2 diabetes (T2D) develop [74]. IR and T2D are highly correlated with lifestyle, in particular with overweightness and obesity. T2D incidence is approximately 90% lower in middle-age women with a normal weight, who exercise regularly and eat a diet rich in cereal fiber and in poly-unsaturated fats and poor in saturated and *trans* fats [75]. Insulin resistance, independent of obesity, strongly correlates with PCOS, a common endocrinology disorders that is estimated to affect between 5% and 10% of reproductive age women [76,77]. This pathology is often characterized by some aberrations in the secretion of gonadotropins and, in particular, with high levels of LH (luteinizing hormone) [78]. Moreover, insulin, through its own receptor, has been demonstrated to have direct effect on steroidogenesis in the ovaries [79]. *In vitro* studies using theca cells from mammalian ovaries have shown that insulin has dose-dependent effects on cell proliferation, steroid production, and expression of genes, such as STAR (steroidogenic acute regulatory protein), CYP11A1 (cytochrome P450 family 11 subfamily A member 1), and CYP17A1 (cytochrome P450 family 17 subfamily A member 1), overall promoting steroidogenesis [80,81,82,83]. In normal, theca cells, insulin, in synergy with LH stimulates CYP17A1 gene expression and the 17α-hydroxylase activity of its product [84], a key enzyme in the regulation of androgen biosynthesis [85], but the molecular mechanism involved in this effect have not been completely elucidated and many conflicting results exist [84,86]. Under physiological conditions, insulin acts as a co-gonadotrophin in theca cells; on the other hand, hyperinsulinemia potentiates gonadotropin-stimulated ovarian androgen synthesis [87,88]. Additionally, in PCOS there is selective insulin resistance on the ovary, as reported for other tissues: insulin action on steroidogenesis is preserved, while insulin action on glucose metabolism is significantly decreased in granulosa-lutein cells from classical PCOS patients [89]. Furthermore, insulin inhibits the hepatic synthesis of sex hormone-binding globulin (SHBG), thus increasing free testosterone levels [87]. All the treatments aimed at reducing insulin levels, such as weight loss and insulin sensitizers, improve female reproductive health.

On the other hand, type 1 diabetes (T1D) is a condition in which pancreatic β-cells destruction leads to absolute insulin deficiency [90]. Hypogonadotropic hypogonadism is present in women with uncontrolled type 1 diabetes [91] and, despite a significant improvement in the therapy for T1D, patients still experience abnormalities in pubertal development, menstrual cycle and menopause age [92]. Experimental evidence further supported the clinical data showing that insulin plays an important role in the regulation of female fertility. Animal models characterized by severe insulinopenia, which mimic T1D, were obtained by treatment with streptozotocin (STZ); indeed, STZ causes rapid and selective elimination of pancreatic β cells [93]. Both STZ males and females exhibit a hypogonadotropic state, characterized by low levels of gonadotropins and sex steroids and by reduced LH pulsatility [94,95,96]. Central insulin administration in STZ treated rats induce normal LH surge, despite the maintenance of peripheral diabetes revealing that insulin action on reproduction occurs at multiple levels [97]. Indeed, insulin receptors (IRs) are located in the CNS in many areas involved in the control of reproduction such as the arcuate, the ventromedial hypothalamic nucleus and the preoptic area [98,99]. Additionally, genetically engineered mice, with a specific deletion of insulin receptor in the CNS, display hypogonadotropic hypogonadism [99]. It appears thus clear that insulin has a great impact of female fertility acting both centrally and in the periphery.

### 5.2. Adipokines

Adipose tissue participates in the regulation of energy homeostasis by acting as an endocrine organ. Indeed, it produces a series of adipokines, which comprises leptin, adiponectin, resistin, tumor necrosis factor-α, interleukin-6, apelin, vaspin, visfatin, hepcidine, chemerin, omentin and many others [100,101,102]. Many of these factors interact with the signal of insulin and could thus indirectly affect reproduction [101]. For others, a clear and direct mechanism affecting fertility has been described; in particular, many studies focused on adiponectin and leptin.

Adiponectin concentration correlates negatively with visceral fat mass in mammals: higher plasmatic concentrations are usually detected in women as compared to men [103] and low levels are generally observed in obese patients [104]. Adiponectin exerts insulin-sensitizing, anti-atherosclerosis and anti-inflammatory actions [105,106,107]; indeed, adiponectin participates in many metabolic processes by modulating insulin sensitivity as well as glucose and lipid metabolism [108]. It seems to have insulin-like effects on target tissues and it was demonstrated to play a role in the regulation of female reproductive functions [101]; indeed, mammalian ovary, uterus and placenta express adiponectin receptor (AdipoR1 and R2) [101,109]. *In vitro* and *in vivo* studies support this view, showing that, in the ovary, adiponectin stimulates steroidogenesis by granulosa cells [109,110] and may play a role in pre-implantation embryo development and uterine receptivity [107]. Adiponectin effect on reproduction could be, at least in part, mediated by its action in the CNS: adiponectin mRNA and AdipoR1 and R2 are highly present in human pituitary [111], where studies conducted in cycling pigs demonstrated that receptors levels vary in response to estrous cycle progression [112,113]. In particular, adiponectin was demonstrated to decrease, in pituitary cell culture, GnRH receptor mRNA and LH release [114]; at this level, adiponectin has been proposed to be involved in an autocrine/paracrine control of pituitary gonadotrophs [112]. Finally, polymorphisms in the human adiponectin precursor gene have been associated with pre-eclampsia and PCOS, and low concentrations of this hormone were found in PCOS patients [101,115,116,117]. Thus, as adiponectin receptors are present and active in many reproductive tissues and given the low levels of the hormone in obese patients, adiponectin could represent an important link between the reproductive system and the adipose tissue [118].

Leptin is produced by the adipose tissue in proportion to the amount of triglycerides stored [119]. Circulating levels of this hormone in humans are highly correlated to BMI and increased markedly in obese subjects [120]. Under physiological conditions, leptin functions to maintain energy homeostasis by reducing food intake, regulating pancreatic islet cells, and fat stores; in obese subjects leptin resistance results in a dysfunctional energetic state [102]. Leptin plasmatic concentrations show sexual dimorphism due, at least in part, to the fact that estrogens promote while androgens suppress leptin synthesis [121,122]. This hormone has been proposed to play a role in the neuroendocrine adaptation to starvation [123]. Low leptin serum concentrations were observed in amenorrheic athletes and in women with anorexia nervosa and could, at least in part, explain the hypothalamic amenorrhea affecting those patients [124]. Indeed, leptin exerts its effects on reproduction acting at multiple sites [125]. In the hypothalamus, it facilitates GnRH secretions through indirect mechanism [126]. Leptin deficient female mice are infertile with low levels of gonadotropins and sex steroids [127,128] and leptin treatment, but not weight loss, corrects sterility [129]. Additionally, individuals with rare leptin deficiency or leptin receptor mutations have hypogonadotropic hypogonadism [128], and leptin administration improves ovarian function in women with hypothalamic amenorrhea [128,130]. Leptin also has a role in ovary and follicular development: in particular, high concentrations of this hormone, such as those observed in obese patients, interfere with estradiol production and oocyte maturation [131]. Leptin receptors have been identified in granulosa, theca and interstitial cells of human ovary, and leptin itself is expressed in human and rat ovaries, where its levels fluctuate across the estrous cycle [132,133,134]. Several *in vitro* and *in vivo* studies demonstrated that the treatment with medium-high physiological doses of this hormone, to resemble those observed in obese patients, inhibited steroidogenesis in human granulosa and theca cells leading to a marked decline in the number of ovulated oocytes [135,136,137]; on the other hand, another study shows that leptin treatment impairs the ovulatory process without affecting steroid levels [138]. Studies on leptin levels in PCOS patients lead to contradictory results. Several investigations reported an increased leptin concentration in PCOS and hypothesized a direct involvement of this hormone in the etiology of the pathology [139,140,141,142,143,144,145,146]. Others find no differences in leptin levels between subjects with or without PCOS [147,148,149,150,151,152,153]: this incongruity could be due to sample sizes, type of sample collection and the heterogeneous nature of the pathology.

In conclusion, it has been hypothesized that very low leptin concentration, as a signal of energy insufficiency, would suppress the reproductive function at the HPG level, while elevated leptin levels, such as those observed in obesity, may have direct inhibitory effects on the gonads.

### 5.3. AMPK, a Molecule Mediating the Signalling of Insulin and Adipokines

Many of the metabolic effects induced by the signals discussed above are mediated by an important molecular player: AMP-activated protein kinase (AMPK). AMPK is a serine/threonine heterotrimeric kinase, sensitive to the AMP: ATP ratio, activated by an increasing AMP concentration and by the upstream kinases including liver kinase B1 (LKB1) and calcium/calmodulin kinase (CaMKK) [154,155]. AMPK is activated in pathophysiological situations (exercise, stress), by metabolic hormones (leptin and adiponectin) or by pharmacological and natural agents (such as metformin, resveratrol and berberine) [156]. It functions as an intracellular energy sensor that switches off ATP-consuming pathways (anabolic, such as protein, glycogen and sterol synthesis) and switches on ATP-producing pathways, such as glycolysis, glucose uptake, mitochondrial biogenesis and fatty acid oxidation [157,158]. The AMPK pathway was viewed primarily as a sensor and regulator of energy balance at cellular level; however, in mammals, it has been demonstrated to be involved in the whole body regulation of energy metabolism responding to both nutritional and hormonal signals in the CNS as well as peripherally [159]. In the central nervous system, hypothalamic AMPK plays a critical role in hormonal and nutrient-derived anorexigenic and orexigenic signals and in energy balance: dominant negative AMPK expression in the hypothalamus is sufficient to reduce food intake and body weight, whereas constitutively active AMPK increases both of them [160]. In the hypothalamus, AMPK mediates the signaling of leptin, insulin and adiponectin. Indeed, in contrast to the periphery, the anorexigenic hormone, leptin, and also insulin inhibit hypothalamic AMPK activity, thus reducing food intake [160,161,162]. On the other hand, the opposite effect has been observed with adiponectin [163]: adiponectin-KO mice showed decreased AMPK activity in the arcuate, decreased food intake, and increased energy expenditure, exhibiting resistance to high-fat-induced obesity [163].

AMPK is ubiquitously expressed and plays a central role in peripheral organs metabolism such as skeletal muscle, liver and fat, integrating the signaling of leptin, insulin and adiponectin [164,165,166]. In skeletal muscle, leptin causes a rapid activation of AMPK thus promoting fatty acid oxidation and energy expenditure [167], the same effect has been observed also in the liver: leptin or leptin-receptor knock out mice showed a reduced AMPK hepatic activity [168]. Adiponectin, through the binding to its receptors, stimulates processes that provoke an increase in AMP and mice treated with adiponectin have enhanced AMPK activation in both skeletal muscle and liver [169,170], thus promoting fatty acid oxidation, glucose uptake and inhibiting glucose production [169,170].

AMPK certainly has a relevant role in the regulation of reproductive function: centrally, its activation by adiponectin has been demonstrated to participate in the regulation of GnRH and LH secretion in response to energy status, in hypothalamic and pituitary cell lines. Several studies have also demonstrated that AMPK is expressed in the gonads [171,172,173] and could play a key role in linking reproductive function with energy balance. Indeed, AMPK is present in granulosa and theca cells, oocytes and corpora lutea in different species: birds [174,175], mammals [171,176,177,178] and humans [179]. AMPK was demonstrated to participate in the control of gonad steroidogenesis and germinal cell maturation but also cell proliferation and survival, polarity, formation, and maintenance of cellular junctional complexes, and cytoskeletal dynamics [180]. Even if no studies have shown a role of AMPK in ovarian steroidogenesis *in vivo*, this has been largely demonstrated *in vitro*. AMPK activators, such as metformin, inhibit the secretion of progesterone and/or estradiol by granulosa cells in mammals [171,176]. In rats, AMPK activation induced by metformin does not reduce aromatase expression and estradiol production but it decreases progesterone synthesis and the expression of different proteins involved in steroidogenesis [181]. In human granulosa cells, metformin also decreases androgen synthesis, by directly inhibiting CYP17A1 activity [182].

## 6. Nutrients as Signaling Molecules

The ability to sense nutrients and to adapt physiological responses to nutrient availability and characteristics allowed organisms to increase their opportunities to survive and to expand their progenies: in one word, to evolve. This ability became relevant for female mammals, in particular with placenta advent: energy metabolism and fertility started to be tightly connected and reciprocally regulated to adapt energy production and storage to the requirements of reproduction [6]. It appears thus clear that nutrients cannot be merely considered a source of energy but they exert a bioactive role; indeed, nutrients represent the main signals for organisms, which behavior cannot disregard from the environment because they must take advantage of the available nutritional resources. According to this view, an increasing amount of literature demonstrates the relevance of the food-dependent modulation of nuclear receptors activity, which has been established and improved throughout evolution.

Nutrients ability in modulating the activity of nuclear receptors mainly occurs through the regulation of signal transduction of the respective regulated pathways. For example, it is well known that fatty acids are the natural inducers of PPARs (peroxisome proliferator-activated receptors alpha, beta, and gamma), nuclear receptors that regulate the transcription of genes involved in cellular differentiation, development, metabolism, and tumorigenesis [183,184,185,186]. Like fatty acids for PPARs, other classes of nutrients may act as agonists of other transcription factors, such as glucose for liver X receptor alpha (LXRα) [187] or amino acids for ERα [6]. Furthermore, some pathways are greatly inter-connected, as demonstrated by different studies highlighting the relevance of the cross-talk between LXRα and PPARα for the modulation of hepatic lipogenesis [188] or between LXRα and farnesoid X receptor (FXR), which regulates bile acids, sterols and fatty acids, which, in turn, are the activators of FXR, LXRα and PPARα, respectively [189]. All these nuclear receptors have a pivotal role for female fertility [190,191,192,193,194,195,196], thus supporting the possibility that specific class of nutrients may contribute to the coordination of energy metabolism and fertility acting as signaling molecules on these receptors.

### 6.1. Poly-Unsaturated Fatty Acids and Female Fertility

In women, the reproductive process and its success are affected by the trend in postponing childbearing, typical in the Western societies [197]. In fact, over the past century, the reproductive lifespan of women has not proportionally increased with the increased woman’s life expectancy [198], as women fertility precipitously declines after the age of 35 [199]. The discrepancy between the overall and the reproductive lifespan of women is more pronounced today than ever before and could be partially related to the changes in the human diet over the past 100 years, most notably with regard to the type and amount of fat consumed [200,201].

In Western diets, the daily caloric intake of fatty acids was estimated around 30%–35%, a value that far exceeds the nutritional requirements [202,203]. Some specific classes of fatty acids, such as the poly-unsaturated (PUFA) omega-3 (*n*-3) and omega-6 (*n*-6) fats, need to be provided with the diet, as they are indispensable for different biological processes, including growth, brain development and reproduction and animals are not able to synthesize them [204,205,206]. Different studies highlighted the beneficial effects exerted by PUFA on metabolic parameters when compared to other type of fatty acids, in particular *trans* fatty acids (TFA) and saturated fatty acids (SFA), and to a lesser extent mono-unsaturated fatty acids (MUFA) [207,208,209,210,211]. However, the beneficial effects exerted by PUFA in counteracting metabolic diseases could be influenced by the type of PUFA, as the optimal *n*-6:*n*-3 PUFA *ratio* changes depending on the pathophysiological condition [212]. Furthermore, other studies have downsized the beneficial metabolic effects of PUFA over other type of fats, in particular MUFA [213].

In women, consumption of TFA instead of MUFA or PUFA is positively associated with ovulatory infertility, independent of age, BMI, lifestyle and hormonal levels [4]. At the mechanistic level, it has been proposed that the detrimental effects of TFA on fertility could be due to their different ability to bind PPARγ and down-regulate its expression [214,215,216]. Furthermore, the higher intake of TFA has been associated with altered metabolic parameters, such as insulin resistance [217], risk of T2D [218], and inflammatory markers [219,220], which may negatively impair ovarian functions. Replacing TFA with MUFA has been demonstrated to be associated with a lower risk of ovulatory infertility [4], although these conclusions could be only partially ascribable to the changes in dietary fats and could be further affected by other nutritional factors, such as the source of proteins (vegetable *vs.* animal), the higher intake of high-fiber, low glycemic carbohydrates, high-fat dairy products, and other micronutrients [4].

In addition, several studies demonstrated that a higher intake of PUFA [221,222,223,224] instead of TFA [225], SFA [225] or MUFA [226] has been associated with the improvement of metabolic and hormonal characteristics in women with PCOS [221]. However, other studies performed in PCOS women failed to confirm some of these beneficial effects [221] and have found that a diet enriched in MUFA instead of PUFA could be more useful in ameliorating the metabolic profile and consequently the fertility rate [227].

The relative *n*-6 and *n*-3 PUFA content has been significantly changed: in the Western diet, the *ratio* of *n*-6 to *n*-3 PUFA ranges from 10:1 to 25:1, greatly diverging from the 1:1 *ratio* typical of the primitive diet, from which our genetic constitution was selected. This change in diet composition, over the last 100 years, has been associated to decreased fertility rates in women over the age of 35 [228]. At the mechanistic level, the proportion of different PUFAs in the diet significantly influences prostaglandin (PG) [229,230,231] synthesis and ovarian steroidogenesis, both having crucial role in the reproductive process [232,233].

PUFA, in particular arachidonic acid (AA) and its metabolites, influence steroidogenesis in female mammals by exerting direct effects on specific enzymes such as STAR and CYP11A1, and on the regulation of PG synthesis [229]. PUFA may also alter the function of nuclear receptors such as LXRα [234] and PPARs [230], by influencing the transcription of their target genes involved in PG synthesis and ovarian steroidogenesis [235]. Furthermore, the amount and type of dietary PUFA might affect several metabolic pathways and the consequent metabolic impairment might cause negative reproductive outcomes [236]. The amount and the type of dietary PUFA might affect different stages of the reproduction, not only ovarian steroid synthesis [237], but also oocyte maturation [238], uterine activity [239], pregnancy [240] and labor [241].

The association between the increased *n*-6:*n*-3 PUFA *ratio* in Western diet and the reduced fertility lifespan in women suggests that a diet enriched in *n*-3 PUFA could be useful in counteracting the relative excess of *n*-6 PUFA and could thus improve the reproductive success. Although some studies seem to confirm this hypothesis [242,243,244], other works do not sustain any positive effect of PUFA on female reproduction [245,246] or even report health problems associated to chronic treatments [241]. This discrepancy could rely on the fact that some investigations were underpowered, lack appropriate controls or were directed only on a specific subset of PUFA [240]. In addition, the specific mechanisms underpinning the positive impact of *n*-3 PUFA on the female reproductive axis remain to be fully elucidated.

In female mammals, diets enriched in *n*-3 PUFA enhance plasmatic levels of 17β-estradiol (E2) [232,247,248], thereby leading to increased secretion of GnRH and triggering the LH surge. The mechanism behind the enhanced E2 synthesis is still unclear; however, several explanations have been suggested: PUFA supplementation might enhance plasma steroid concentrations by increasing the availability of lipoprotein–cholesterol, by modulating PG synthesis, or by directly stimulating ovarian steroidogenesis [249,250]. Conversely, other studies [251,252] suggested that the greater and long-lasting elevation of E2 levels in female mammals consuming *n*-3 PUFA could be due to the inhibitory effects exerted by *n*-3 PUFA on the metabolism of steroid hormones in the liver [253,254,255].

The delay in E2 surge and in the consequent positive feedback of E2 on hypothalamus and pituitary could explain the tendency for a delay in LH peak and ovulation observed in females supplemented with PUFA [256]. Furthermore, dietary PUFA could influence ovulation timing by acting on PG synthesis and PG-mediated changes in LHRH release [257,258].

Some studies showed that the content of E2 and the 17β-estradiol/progesterone (E2/P4) *ratio*, a reliable indicator of follicles health [259,260], are increased in the pre-ovulatory follicles of females supplemented with PUFA [261]. All these hormonal changes contribute to the increased number and size of pre-ovulatory follicles and may be beneficial for ovarian function.

In PCOS women, the supplementation of *n*-3 PUFA ameliorates both the metabolic and hormonal parameters (in particular, by decreasing the levels of androgens) [223,226], even if others studies failed to unravel significant changes in the regulation of HPG axis [221]. Conversely, studies done in a rat model of PCOS demonstrated that dietary *n*-3 PUFA are able to increase the mean of FSH and significantly decrease the testosterone levels [262].

### 6.2. Carbohydrates and Sugars and Female Fertility

The strong relationship between decreased insulin sensitivity and women infertility observed in diabetics and PCOS women [263] suggests that the amount and quality of carbohydrates in diet might influence reproductive functions. The existence and the mechanism of the correlation between sugars and reproduction, in healthy premenopausal women, are far from being fully elucidated: in the literature, many conflicting data are present.

On the one hand, some studies demonstrated that the quality of quantity of dietary carbohydrates might be associated with ovulatory infertility among nulliparous women [264]. The mechanism could be mainly ascribable to reduced insulin sensitivity that leads to increased free IGF-I and androgen levels [265], thus reproducing some clinical features typical of PCOS [78]. The impact of carbohydrates on the HPG axis has been suggested in a longitudinal study done in women, where a low-fat, high-carbohydrate diet decreased significantly the blood levels of E2 and P4 and increased the levels of FSH and the ratio FSH:E2, independent of age and weight [266]. The lower E2 levels and the longer menstrual cycles observed in women subjected to this diet partly reflect the changes in the years that precede the menopause [267]. On the other hand other studies did not found any significant association between dietary intake of these macronutrients and plasma sex steroid levels [268,269]. These discrepancies could rely on the different protocol adopted (intake and sources of carbohydrates, length of the treatment) and on the magnitude of the study. The main effects of high intake of carbohydrates could be mainly mediated by insulin and its signalling pathway, thereby affecting the HPG axis (see also Section 5.1). A captivating explanation suggests that the impairment of the ovulatory process is not due to the increased carbohydrate intake *per se*, but could rather be linked to the fact that the increasing carbohydrate intake is at the expense of natural fats, which exert a beneficial effect on ovulatory function [270].

Even if the consumption of carbohydrates and sugars—particularly fructose in liquids, such as in sugar-sweetened beverage (SSB)—is decreasing [271], mean intakes among premenopausal women continue to exceed recommendations and might affect the reproductive process. Diets high in carbohydrates/sugars lead to dyslipidemia and insulin resistance, thereby causing hormonal and ovulatory disorders, however very few studies have assessed the effects of energy containing beverages on hormonal levels and ovulatory function in premenopausal women. One study showed that high carbohydrate intake is associated with an increased risk of anovulatory infertility: dietary glycemic index is positively related to this condition in a cohort of apparently healthy women [264]. However, other studies did not found any association between SSB and premenopausal reproductive hormones [272,273] or, even if they showed an association between sugars intake and elevated follicular free and total estradiol, these altered hormone levels do not interfere with ovulation among healthy premenopausal women without ovulatory disorders [274,275].

This discrepancy might, once again, be due to limitations in the studies, such as small sample sizes and/or inadequate assessment of nutritional and hormonal variables.

### 6.3. Proteins, Amino Acids and Female Reproduction

Few studies done in overweight women with PCOS [276,277] showed that diet enriched in proteins (30% *vs.* 15% of total energy) improved menstrual cyclicity by reducing circulating androgens levels and improving insulin sensitivity as a consequence of weight loss. In these studies, the increased protein intake had no effect on reproductive function *per se* indeed, the small improvements in menstrual cyclicity seems to be ascribable to a greater insulin sensitivity associated to a reduced carbohydrates intake (replaced by proteins) rather than an increased dietary protein intake.

The Nurses’ Health Study II (NHS II) highlighted the association between animal proteins intake and increased risk of ovulatory infertility in a cohort of healthy women [278]; conversely, the consumption of proteins from vegetable sources instead of carbohydrates or animal protein was associated with a substantially lower risk of ovulatory infertility, at least among women older than 32 years [278], thus suggesting that protein intake may differently affect female fertility depending on the protein source. Although the biological mechanisms responsible for this association have not been identified, evidence indicates that the benefits associated to a diet enriched in proteins from vegetable instead of animal sources might rely on the increased insulin sensitivity [279,280] and might be associated to changes in circulating free IGF-I levels [281].

These evidences seem to contradict other studies showing an association between vegetarian/vegan diets and menstrual disturbances [282,283,284,285,286,287]. However, most of the studies showing a correlation between these diets and menstrual disturbances were performed in athletes, in which the elevated energy expenditure consequent to physical activity may be the main cause leading to reproductive alterations. Similarly, the menstrual disturbances observed in vegetarian women, who are generally leaner and lighter than non-vegetarian ones [288,289,290], may be due to reduced energy availability and increased physical activity [290] rather than a deficiency in dietary protein intake.

The reproductive effects of vegetarian/vegan diets have not been fully elucidated. Very few studies showed impairments in the reproductive parameters among vegetarian women in comparison to non-vegetarians [285]. Others did not support any vegetarian diet dependent difference related to the reproductive process [291]. Similarly, although some studies showed altered reproductive outcomes [292,293,294,295,296], others did not support any significant differences between vegetarian and non-vegetarian diet in relation to pregnancy outcomes [297,298,299]. These discrepancies may be due to the paucity and limitations of the studies; indeed, they often did not take into account the possible effects dependent upon changes in others macronutrient classes such as fat and fiber and the lifestyle of vegetarian *vs.* non-vegetarian women. Additionally, in these studies, the number of observations is often restricted to 1–2 menstrual cycles. Furthermore, some studies lack of significance because they considered underpowered and not randomized groups. Finally, others studies performed in larger populations could be affected by peculiar lifestyles (such as abstention from drugs, alcohol, tobacco, and caffeine-containing beverages) [293], thus making impossible to elucidate the specific role of the vegetarian/vegan diet.

In some studies performed in female mammals, the impact of dietary proteins was associated with changes in HPG axis regulation: in particular, a higher intake of proteins was shown to enhance GnRH-induced LH release [300,301]. Conversely, in other studies, diets deficient in proteins delay puberty in female ruminants but does not impair the synthesis and processing of LHRH in the brain neurons and synthesis of LH in pituitary cells [302]. Furthermore, most studies done in female ruminants showed that higher intake of proteins increases ovarian activity by non LH-mediated pathways, but acting at a local level, through changes in insulin [303] or IGF system, by enhancing the sensitivity of follicles toward FSH and regulating oocyte quality [304]. According to this data, we demonstrated that a diet enriched in amino acids is able to partly counteract the block of estrous cycle progression in mouse females subjected to 40% calorie restriction: this effect is mediated by changes in IGF-1 levels that we showed to be dependent on the hepatic activity of estrogen receptor alpha (ERα) [10].

Dietary intake of proteins may affect the circulating levels of P4, although different studies led to opposite results and others showed no changes [305,306,307]. The discrepancy could be related to the different protein-enriched diets adopted in these studies (different percentage of proteins or different source of proteins). Another explanation for these conflicting responses could be ascribable to the difference in the lactation status: high dietary proteins reduced plasma P4 concentrations in lactating [305,306,308], but not in non-lactating female mammals [308,309,310,311].

In women under physiological conditions, amino acids levels fluctuate during the menstrual cycle and, in particular, decrease in the luteal phase [312,313,314]. It has been suggested that the decreased plasma amino acid levels reflect an increased utilization and could be due to the raised levels of P4 and E2 [312,315,316]. Although the dietary intake of proteins has not been evaluated during the menstrual cycle progression, the decreased in plasma amino acid levels measured during the luteal phase could be the consequence of the increased physiological demands of metabolic intermediates for steroid synthesis by the corpus luteum as well as glycogenesis [317,318], protein synthesis and secretion by the endometrium [319]. This fascinating suggestion might further explain the association between the reduced fertility in women observed nowadays and the decrease in protein intake in the industrialized societies compared to the nutritional environment for which our genetic constitution was selected [200,320].

### 6.4. Food-Associated Endocrine Disrupting Chemicals and Female Fertility

Some naturally-occurring or industrial-derived food components can have adverse effects *per se* and could interfere and impair the signaling pathways regulating the reproductive process. It has been shown that very high doses of genistein, a phytoestrogen present in food in particular in soy milk, could have adverse effects on female reproductive physiology [321] and on pregnancy outcomes [322,323,324]. Similarly, endocrine disrupting chemicals (EDCs) present in food as additives or contaminants [325] were proposed to be associated with altered reproductive functions both in males and females [326,327,328,329,330,331].

Some substances, for their chemical structures, could accumulate in tissues and, once mobilized under energetic imbalance, could exert their action in the whole organism. For example, it has been reported that genistein might accumulate in placenta [332] or in body depots [333], where, once mobilized during fasting, might reach the fetus through the maternal-fetal transfer [334,335,336].

In females, EDCs affect steroidogenesis by acting on the HPG axis [325,337,338,339], impair ovarian development and function [340,341,342,343,344,345,346,347] and cause uterine and ovarian alterations such as endometriosis [348,349,350,351], premature ovarian failure (POF) [352] and PCOS [353].

Although the mechanisms of action of ECDs are difficult to unravel given the complexity of the endocrine system, ECDs were demonstrated to interfere with the ER or the androgen receptor (AR) signaling resulting in synergistic or antagonistic outcomes [354]. Furthermore, ECDs can modify hormones bioavailability by interfering with their synthesis, secretion, transport and metabolism [355,356,357,358,359,360,361].

## 7. The Beneficial Effects of Mediterranean Diet for Women Health

The benefits of the Mediterranean-type diet [362,363] on health [364,365] include a significant reduction of the incidence of metabolic [366,367,368,369,370,371,372], cardiovascular [373,374,375,376,377,378], neurodegenerative diseases [379,380,381,382], and cancer [383,384,385]. In fertile age women, the adherence to the Mediterranean-type diet seems to reduce the risk of weight gain and insulin resistance [386,387,388,389,390,391], thereby increasing the chance of pregnancy, as suggested by a study showing a 40% increase in successful pregnancy among couples undergoing IVF [392]. Furthermore, the adherence to Mediterranean-style dietary pattern was inversely associated with the risk of developing obstetric complications associated with adverse health outcomes for the mother and child, including hypertensive disorders of pregnancy (HDPs) [393], preterm delivery [394], gestational diabetes mellitus [395,396], low intra-uterine size and low birth weight [397]. For these evidences the Mediterranean diet has been suggested as a “preconception diet” for couples undergoing *in vitro* fertilization treatment [392].

Although the studies that specifically evaluate the effects of the Mediterranean-type diet on women fertility are scarce, different studies done on female experimental mammals suggested that the increased intake of vegetable oils that are rich in linoleic acid, an *n*-6 fatty acid, which can only be obtained by the diet, may improve the reproductive process. In fact, linoleic acid and others *n*-6 fatty acids, being precursors of the prostaglandins, might play an important role in the initiation of the menstrual cycle [398], growth, and development of pre antral follicles and ovulation [232,399], as like in the maintenance of pregnancy by optimizing endometrial receptivity [229,400,401]. Some studies showed that consuming *n*-6 PUFA instead of TFA was associated with a reduced risk of ovulatory infertility [402].

On the other hand, other studies seem to disprove these indications reporting that the adherence to Mediterranean diet was not associated with lower incidence of menstrual disturbances [233] or with reduced risk of pregnancy loss [403,404].

## 8. Conclusions

Although different studies suggest that physical activity and a proper diet, in particular the daily intake of different classes of nutrients, could significantly improve reproductive outcomes [4], the identification of a fertility diet is still the “Holy Grail” in female infertility management. The main causes rely on the lack of a full comprehension of the activity and mechanisms of action of many nutrients, along with other variables such as lifestyle, physical activity, and genetic and cultural backgrounds. Unfortunately, we are far from shedding a light on this area of research; first of all because of the paucity of the studies present in the literature. Secondary, it is still difficult to recruit and to persuade healthy volunteers to participate in longitudinal studies based on nutrition and lifestyle interventions. Furthermore, the different protocols adopted during clinical tests often resulted underpowered and not randomized, thus leading to conflicting results. The lack of longitudinal studies focused on the reproductive outcome of diet that could, at the same time, evaluate the differences in lifestyle, physical activity, genetic and cultural backgrounds is a main problem. Additionally, the lack of a common protocol of analysis (the methods used, the parameters and the endpoints evaluated are generally different among the different studies) makes it impossible to integrate the high heterogeneity of data available and currently represent a main hindrance in leading to conclusive results.

Nevertheless, nutrition and lifestyle changes are still one of the most promising and invaluable interventions in preserving human health and women fertility and represent the most captivating challenge that we also have to take up today as Hippocrates, in the 4th century BC, suggested:

“If we could give every individual the right amount of nourishment and exercise, not too little and not too much, we would have found the safest way to health”.

## References

[1] Inhorn M.C., Patrizio P. (2015). Infertility around the globe: New thinking on gender, reproductive technologies and global movements in the 21st century. Hum. Reprod. Update.

[2] Ombelet W., Cooke I., Dyer S., Serour G., Devroey P. (2008). Infertility and the provision of infertility medical services in developing countries. Hum. Reprod. Update.

[3] Templeton A. (2000). Infertility and the establishment of pregnancy—Overview. Br. Med. Bull..

[4] Chavarro J.E., Rich-Edwards J.W., Rosner B.A., Willett W.C. (2007). Diet and lifestyle in the prevention of ovulatory disorder infertility. Obstet. Gynecol..

[5] Hassan M.A., Killick S.R. (2004). Negative lifestyle is associated with a significant reduction in fecundity. Fertil. Steril..

[6] Della Torre S., Benedusi V., Fontana R., Maggi A. (2014). Energy metabolism and fertility: A balance preserved for female health. Nat. Rev. Endocrinol..

[7] Mircea C.N., Lujan M.E., Pierson R.A. (2007). Metabolic fuel and clinical implications for female reproduction. J. Obstet. Gynaecol. Can..

[8] Wade G.N., Schneider J.E. (1992). Metabolic fuels and reproduction in female mammals. Neurosci. Biobehav. Rev..

[9] Shapira N. (2013). Women’s higher health risks in the obesogenic environment: A gender nutrition approach to metabolic dimorphism with predictive, preventive, and personalised medicine. EPMA J..

[10] Della Torre S., Rando G., Meda C., Stell A., Chambon P., Krust A., Ibarra C., Magni P., Ciana P., Maggi A. (2011). Amino acid-dependent activation of liver estrogen receptor alpha integrates metabolic and reproductive functions via IGF-1. Cell Metab..

[11] Essah P.A., Nestler J.E. (2006). The metabolic syndrome in polycystic ovary syndrome. J. Endocrinol. Investig..

[12] Gravholt C.H. (2004). Epidemiological, endocrine and metabolic features in Turner syndrome. Eur. J. Endocrinol..

[13] Wing R.R., Matthews K.A., Kuller L.H., Meilahn E.N., Plantinga P.L. (1991). Weight gain at the time of menopause. Arch. Intern. Med..

[14] Talmor A., Dunphy B. (2015). Female obesity and infertility. Best Pract. Res. Clin. Obstet. Gynaecol..

[15] Chavarro J.E., Toth T.L., Wright D.L., Meeker J.D., Hauser R. (2010). Body mass index in relation to semen quality, sperm DNA integrity, and serum reproductive hormone levels among men attending an infertility clinic. Fertil. Steril..

[16] Schneider J.E. (2004). Energy balance and reproduction. Physiol. Behav..

[17] Stubbins R.E., Holcomb V.B., Hong J., Nunez N.P. (2012). Estrogen modulates abdominal adiposity and protects female mice from obesity and impaired glucose tolerance. Eur. J. Nutr..

[18] Tchernof A., Desmeules A., Richard C., Laberge P., Daris M., Mailloux J., Rheaume C., Dupont P. (2004). Ovarian hormone status and abdominal visceral adipose tissue metabolism. J. Clin. Endocrinol. Metab..

[19] Heine P.A., Taylor J.A., Iwamoto G.A., Lubahn D.B., Cooke P.S. (2000). Increased adipose tissue in male and female estrogen receptor-alpha knockout mice. Proc. Natl. Acad. Sci. USA.

[20] Xu Y., Nedungadi T.P., Zhu L., Sobhani N., Irani B.G., Davis K.E., Zhang X., Zou F., Gent L.M., Hahner L.D. (2011). Distinct hypothalamic neurons mediate estrogenic effects on energy homeostasis and reproduction. Cell Metab..

[21] Olofsson L.E., Pierce A.A., Xu A.W. (2009). Functional requirement of AgRP and NPY neurons in ovarian cycle-dependent regulation of food intake. Proc. Natl. Acad. Sci. USA.

[22] Xu Y., Zhang W., Klaus J., Young J., Koerner I., Sheldahl L.C., Hurn P.D., Martinez-Murillo F., Alkayed N.J. (2006). Role of cocaine- and amphetamine-regulated transcript in estradiol-mediated neuroprotection. Proc. Natl. Acad. Sci. USA.

[23] Asarian L., Geary N. (2007). Estradiol enhances cholecystokinin-dependent lipid-induced satiation and activates estrogen receptor-alpha-expressing cells in the nucleus tractus solitarius of ovariectomized rats. Endocrinology.

[24] Sakurazawa N., Mano-Otagiri A., Nemoto T., Shibasaki T. (2013). Effects of intracerebroventricular ghrelin on food intake and Fos expression in the arcuate nucleus of the hypothalamus in female rats vary with estrous cycle phase. Neurosci. Lett..

[25] Clegg D.J., Brown L.M., Woods S.C., Benoit S.C. (2006). Gonadal hormones determine sensitivity to central leptin and insulin. Diabetes.

[26] Lu K.H., Hopper B.R., Vargo T.M., Yen S.S. (1979). Chronological changes in sex steroid, gonadotropin and prolactin secretions in aging female rats displaying different reproductive states. Biol. Reprod..

[27] D’Eon T.M., Souza S.C., Aronovitz M., Obin M.S., Fried S.K., Greenberg A.S. (2005). Estrogen regulation of adiposity and fuel partitioning. Evidence of genomic and non-genomic regulation of lipogenic and oxidative pathways. J. Biol. Chem..

[28] Lundholm L., Zang H., Hirschberg A.L., Gustafsson J.A., Arner P., Dahlman-Wright K. (2008). Key lipogenic gene expression can be decreased by estrogen in human adipose tissue. Fertil. Steril..

[29] Le May C., Chu K., Hu M., Ortega C.S., Simpson E.R., Korach K.S., Tsai M.J., Mauvais-Jarvis F. (2006). Estrogens protect pancreatic beta-cells from apoptosis and prevent insulin-deficient diabetes mellitus in mice. Proc. Natl. Acad. Sci. USA.

[30] Tiano J.P., Delghingaro-Augusto V., Le May C., Liu S., Kaw M.K., Khuder S.S., Latour M.G., Bhatt S.A., Korach K.S., Najjar S.M. (2011). Estrogen receptor activation reduces lipid synthesis in pancreatic islets and prevents beta cell failure in rodent models of type 2 diabetes. J. Clin. Investig..

[31] Linna M.S., Raevuori A., Haukka J., Suvisaari J.M., Suokas J.T., Gissler M. (2013). Reproductive health outcomes in eating disorders. Int. J. Eat. Disord..

[32] Couse J.F., Korach K.S. (1999). Estrogen receptor null mice: What have we learned and where will they lead us?. Endocr. Rev..

[33] Villa A., Della Torre S., Stell A., Cook J., Brown M., Maggi A. (2012). Tetradian oscillation of estrogen receptor alpha is necessary to prevent liver lipid deposition. Proc. Natl. Acad. Sci. USA.

[34] Shen M., Shi H. (2015). Sex Hormones and Their Receptors Regulate Liver Energy Homeostasis. Int. J. Endocrinol..

[35] Kelly-Weeder S., O’Connor A. (2006). Modifiable risk factors for impaired fertility in women: What nurse practitioners need to know. J. Am. Acad. Nurse Pract..

[36] Chavarro J.E., Gaskins A.J., Afeiche M.C., Tremellen K., Pearce K. (2015). Nutrition and Ovulatory Function. Nutrition, Fertility and Human Reproductive Function.

[37] Cousins A., Freizinger M., Duffy M.E., Gregas M., Wolfe B.E. (2015). Self-report of eating disorder symptoms among women with and without infertility. J. Obstet. Gynecol. Neonatal Nurs..

[38] Gudmundsdottir S.L., Flanders W.D., Augestad L.B. (2014). Menstrual Cycle Abnormalities in Healthy Women With Low Physical Activity: The North-Trondelag Population-based Health Study. J. Phys. Act. Health.

[39] Treasure J., Claudino A.M., Zucker N. (2010). Eating disorders. Lancet.

[40] Leyendecker G., Wildt L. (1984). Pulsatile administration of Gn-RH in hypothalamic amenorrhea. Ups J. Med. Sci..

[41] Devlin M.J., Walsh B.T., Katz J.L., Roose S.P., Linkie D.M., Wright L., Vande Wiele R., Glassman A.H. (1989). Hypothalamic-pituitary-gonadal function in anorexia nervosa and bulimia. Psychiatry Res..

[42] Couzinet B., Young J., Brailly S., Le Bouc Y., Chanson P., Schaison G. (1999). Functional hypothalamic amenorrhoea: A partial and reversible gonadotrophin deficiency of nutritional origin. Clin. Endocrinol..

[43] Warren M.P. (1980). The effects of exercise on pubertal progression and reproductive function in girls. J. Clin. Endocrinol. Metab..

[44] Norman R.J., Noakes M., Wu R., Davies M.J., Moran L., Wang J.X. (2004). Improving reproductive performance in overweight/obese women with effective weight management. Hum. Reprod. Update.

[45] Clark A.M., Ledger W., Galletly C., Tomlinson L., Blaney F., Wang X., Norman R.J. (1995). Weight loss results in significant improvement in pregnancy and ovulation rates in anovulatory obese women. Hum. Reprod..

[46] Loucks A.B. (2003). Energy availability, not body fatness, regulates reproductive function in women. Exerc. Sport Sci. Rev..

[47] ESHRE Capri Workshop Group (2006). Nutrition and reproduction in women. Hum. Reprod. Update.

[48] Rome E.S., Ammerman S. (2003). Medical complications of eating disorders: An update. J. Adolesc. Health.

[49] Kanter R., Caballero B. (2012). Global gender disparities in obesity: A review. Adv. Nutr..

[50] Flegal K.M., Carroll M.D., Kit B.K., Ogden C.L. (2012). Prevalence of obesity and trends in the distribution of body mass index among US adults, 1999–2010. JAMA.

[51] Skinner A.C., Skelton J.A. (2014). Prevalence and trends in obesity and severe obesity among children in the United States, 1999–2012. JAMA Pediatr..

[52] Kopelman P.G. (2000). Obesity as a medical problem. Nature.

[53] Pasquali R. (2006). Obesity, fat distribution and infertility. Maturitas.

[54] Moran L.J., Norman R.J., Teede H.J. (2015). Metabolic risk in PCOS: Phenotype and adiposity impact. Trends Endocrinol. Metab..

[55] Ahmed M.L., Ong K.K., Dunger D.B. (2009). Childhood obesity and the timing of puberty. Trends Endocrinol. Metab..

[56] Lakshman R., Forouhi N., Luben R., Bingham S., Khaw K., Wareham N., Ong K.K. (2008). Association between age at menarche and risk of diabetes in adults: Results from the EPIC-Norfolk cohort study. Diabetologia.

[57] Velie E.M., Nechuta S., Osuch J.R. (2005). Lifetime reproductive and anthropometric risk factors for breast cancer in postmenopausal women. Breast Dis..

[58] Diamanti-Kandarakis E., Bergiele A. (2001). The influence of obesity on hyperandrogenism and infertility in the female. Obes. Rev..

[59] Michalakis K., Mintziori G., Kaprara A., Tarlatzis B.C., Goulis D.G. (2013). The complex interaction between obesity, metabolic syndrome and reproductive axis: A narrative review. Metabolism.

[60] Van der Steeg J.W., Steures P., Eijkemans M.J., Habbema J.D., Hompes P.G., Burggraaff J.M., Oosterhuis G.J., Bossuyt P.M., van der Veen F., Mol B.W. (2008). Obesity affects spontaneous pregnancy chances in subfertile, ovulatory women. Hum. Reprod..

[61] Bellver J., Ayllon Y., Ferrando M., Melo M., Goyri E., Pellicer A., Remohi J., Meseguer M. (2010). Female obesity impairs *in vitro* fertilization outcome without affecting embryo quality. Fertil. Steril..

[62] Bellver J., Melo M.A., Bosch E., Serra V., Remohi J., Pellicer A. (2007). Obesity and poor reproductive outcome: The potential role of the endometrium. Fertil. Steril..

[63] Klop B., Elte J.W., Cabezas M.C. (2013). Dyslipidemia in obesity: Mechanisms and potential targets. Nutrients.

[64] Fujimoto V.Y., Kane J.P., Ishida B.Y., Bloom M.S., Browne R.W. (2010). High-density lipoprotein metabolism and the human embryo. Hum. Reprod. Update.

[65] Grummer R.R., Carroll D.J. (1988). A review of lipoprotein cholesterol metabolism: Importance to ovarian function. J. Anim. Sci..

[66] Mouzat K., Baron S., Marceau G., Caira F., Sapin V., Volle D.H., Lumbroso S., Lobaccaro J.M. (2013). Emerging roles for LXRs and LRH-1 in female reproduction. Mol. Cell. Endocrinol..

[67] Schisterman E.F., Mumford S.L., Browne R.W., Barr D.B., Chen Z., Louis G.M. (2014). Lipid concentrations and couple fecundity: The LIFE study. J. Clin. Endocrinol. Metab..

[68] Miettinen H.E., Rayburn H., Krieger M. (2001). Abnormal lipoprotein metabolism and reversible female infertility in HDL receptor (SR-BI)-deficient mice. J. Clin. Investig..

[69] Della Torre S., Mitro N., Fontana R., Gomaraschi M., Favari E., Recordati C., Lolli F., Quagliarini F., Meda C., Ohlsson C. (2015). The essential role of liver ERα in coupling hepatic metabolism to the reproductive cycle. Cell Rep..

[70] Budak E., Fernandez Sanchez M., Bellver J., Cervero A., Simon C., Pellicer A. (2006). Interactions of the hormones leptin, ghrelin, adiponectin, resistin, and PYY3–36 with the reproductive system. Fertil. Steril..

[71] Comninos A.N., Jayasena C.N., Dhillo W.S. (2014). The relationship between gut and adipose hormones, and reproduction. Hum. Reprod. Update.

[72] Banting F.G., Best C.H., Collip J.B., Campbell W.R., Fletcher A.A. (1922). Pancreatic Extracts in the Treatment of Diabetes Mellitus. Can. Med. Assoc. J..

[73] American Diabetes Association (1997). Consensus Development Conference on Insulin Resistance. 5–6 November 1997. Diabetes Care.

[74] Donath M.Y., Shoelson S.E. (2011). Type 2 diabetes as an inflammatory disease. Nat. Rev. Immunol..

[75] Hu F.B., Manson J.E., Stampfer M.J., Colditz G., Liu S., Solomon C.G., Willett W.C. (2001). Diet, lifestyle, and the risk of type 2 diabetes mellitus in women. N. Engl. J. Med..

[76] Diamanti-Kandarakis E., Kouli C.R., Bergiele A.T., Filandra F.A., Tsianateli T.C., Spina G.G., Zapanti E.D., Bartzis M.I. (1999). A survey of the polycystic ovary syndrome in the Greek island of Lesbos: Hormonal and metabolic profile. J. Clin. Endocrinol. Metab..

[77] Asuncion M., Calvo R.M., San Millan J.L., Sancho J., Avila S., Escobar-Morreale H.F. (2000). A prospective study of the prevalence of the polycystic ovary syndrome in unselected Caucasian women from Spain. J. Clin. Endocrinol. Metab..

[78] Ehrmann D.A. (2005). Polycystic ovary syndrome. N. Engl. J. Med..

[79] Nestler J.E., Jakubowicz D.J., de Vargas A.F., Brik C., Quintero N., Medina F. (1998). Insulin stimulates testosterone biosynthesis by human thecal cells from women with polycystic ovary syndrome by activating its own receptor and using inositolglycan mediators as the signal transduction system. J. Clin. Endocrinol. Metab..

[80] Morley P., Calaresu F.R., Barbe G.J., Armstrong D.T. (1989). Insulin enhances luteinizing hormone-stimulated steroidogenesis by porcine theca cells. Biol. Reprod..

[81] Campbell B.K., Scaramuzzi R.J., Webb R. (1995). Control of antral follicle development and selection in sheep and cattle. J. Reprod. Fertil. Suppl..

[82] Mamluk R., Greber Y., Meidan R. (1999). Hormonal regulation of messenger ribonucleic acid expression for steroidogenic factor-1, steroidogenic acute regulatory protein, and cytochrome P450 side-chain cleavage in bovine luteal cells. Biol. Reprod..

[83] Young J.M., McNeilly A.S. (2010). Theca: The forgotten cell of the ovarian follicle. Reproduction.

[84] Munir I., Yen H.W., Geller D.H., Torbati D., Bierden R.M., Weitsman S.R., Agarwal S.K., Magoffin D.A. (2004). Insulin augmentation of 17alpha-hydroxylase activity is mediated by phosphatidyl inositol 3-kinase but not extracellular signal-regulated kinase-1/2 in human ovarian theca cells. Endocrinology.

[85] Marsh C.A., Auchus R.J. (2014). Fertility in patients with genetic deficiencies of cytochrome P450c17 (CYP17A1): Combined 17-hydroxylase/17,20-lyase deficiency and isolated 17,20-lyase deficiency. Fertil. Steril..

[86] Nelson-Degrave V.L., Wickenheisser J.K., Hendricks K.L., Asano T., Fujishiro M., Legro R.S., Kimball S.R., Strauss J.F., McAllister J.M. (2005). Alterations in mitogen-activated protein kinase kinase and extracellular regulated kinase signaling in theca cells contribute to excessive androgen production in polycystic ovary syndrome. Mol. Endocrinol..

[87] Diamanti-Kandarakis E., Dunaif A. (2012). Insulin resistance and the polycystic ovary syndrome revisited: An update on mechanisms and implications. Endocr. Rev..

[88] Tosi F., Negri C., Perrone F., Dorizzi R., Castello R., Bonora E., Moghetti P. (2012). Hyperinsulinemia amplifies GnRH agonist stimulated ovarian steroid secretion in women with polycystic ovary syndrome. J. Clin. Endocrinol. Metab..

[89] Wu X.K., Zhou S.Y., Liu J.X., Pollanen P., Sallinen K., Makinen M., Erkkola R. (2003). Selective ovary resistance to insulin signaling in women with polycystic ovary syndrome. Fertil. Steril..

[90] Daneman D. (2006). Type 1 diabetes. Lancet.

[91] Griffin M.L., South S.A., Yankov V.I., Booth R.A., Asplin C.M., Veldhuis J.D., Evans W.S. (1994). Insulin-dependent diabetes mellitus and menstrual dysfunction. Ann. Med..

[92] Codner E., Merino P.M., Tena-Sempere M. (2012). Female reproduction and type 1 diabetes: From mechanisms to clinical findings. Hum. Reprod. Update.

[93] King A.J. (2012). The use of animal models in diabetes research. Br. J. Pharmacol..

[94] Katayama S., Brownscheidle C.M., Wootten V., Lee J.B., Shimaoka K. (1984). Absent or delayed preovulatory luteinizing hormone surge in experimental diabetes mellitus. Diabetes.

[95] Chandrashekar V., Steger R.W., Bartke A., Fadden C.T., Kienast S.G. (1991). Influence of diabetes on the gonadotropin response to the negative feedback effect of testosterone and hypothalamic neurotransmitter turnover in adult male rats. Neuroendocrinology.

[96] Spindler-Vomachka M., Johnson D.C. (1985). Altered hypothalamic-pituitary function in the adult female rat with streptozotocin-induced diabetes. Diabetologia.

[97] Kovacs P., Parlow A.F., Karkanias G.B. (2002). Effect of centrally administered insulin on gonadotropin-releasing hormone neuron activity and luteinizing hormone surge in the diabetic female rat. Neuroendocrinology.

[98] Plum L., Schubert M., Bruning J.C. (2005). The role of insulin receptor signaling in the brain. Trends Endocrinol. Metab..

[99] Bruning J.C., Gautam D., Burks D.J., Gillette J., Schubert M., Orban P.C., Klein R., Krone W., Muller-Wieland D., Kahn C.R. (2000). Role of brain insulin receptor in control of body weight and reproduction. Science.

[100] Wozniak S.E., Gee L.L., Wachtel M.S., Frezza E.E. (2009). Adipose tissue: The new endocrine organ? A review article. Dig. Dis. Sci..

[101] Campos D.B., Palin M.F., Bordignon V., Murphy B.D. (2008). The “beneficial” adipokines in reproduction and fertility. Int. J. Obes..

[102] Mitchell M., Armstrong D.T., Robker R.L., Norman R.J. (2005). Adipokines: Implications for female fertility and obesity. Reproduction.

[103] Chakraborti C.K. (2015). Role of adiponectin and some other factors linking type 2 diabetes mellitus and obesity. World J. Diabetes.

[104] Arita Y., Kihara S., Ouchi N., Takahashi M., Maeda K., Miyagawa J., Hotta K., Shimomura I., Nakamura T., Miyaoka K. (1999). Paradoxical decrease of an adipose-specific protein, adiponectin, in obesity. Biochem. Biophys. Res. Commun..

[105] Kadowaki T., Yamauchi T. (2005). Adiponectin and adiponectin receptors. Endocr. Rev..

[106] Hug C., Lodish H.F. (2005). The role of the adipocyte hormone adiponectin in cardiovascular disease. Curr. Opin. Pharmacol..

[107] Kim S.T., Marquard K., Stephens S., Louden E., Allsworth J., Moley K.H. (2011). Adiponectin and adiponectin receptors in the mouse preimplantation embryo and uterus. Hum. Reprod..

[108] Lau C.H., Muniandy S. (2011). Novel adiponectin-resistin (AR) and insulin resistance (IRAR) indexes are useful integrated diagnostic biomarkers for insulin resistance, type 2 diabetes and metabolic syndrome: A case control study. Cardiovasc. Diabetol..

[109] Chabrolle C., Tosca L., Rame C., Lecomte P., Royere D., Dupont J. (2009). Adiponectin increases insulin-like growth factor I-induced progesterone and estradiol secretion in human granulosa cells. Fertil. Steril..

[110] Ledoux S., Campos D.B., Lopes F.L., Dobias-Goff M., Palin M.F., Murphy B.D. (2006). Adiponectin induces periovulatory changes in ovarian follicular cells. Endocrinology.

[111] Psilopanagioti A., Papadaki H., Kranioti E.F., Alexandrides T.K., Varakis J.N. (2009). Expression of adiponectin and adiponectin receptors in human pituitary gland and brain. Neuroendocrinology.

[112] Kiezun M., Maleszka A., Smolinska N., Nitkiewicz A., Kaminski T. (2013). Expression of adiponectin receptors 1 (AdipoR1) and 2 (AdipoR2) in the porcine pituitary during the oestrous cycle. Reprod. Biol. Endocrinol..

[113] Thundyil J., Pavlovski D., Sobey C.G., Arumugam T.V. (2012). Adiponectin receptor signalling in the brain. Br. J. Pharmacol..

[114] Rodriguez-Pacheco F., Martinez-Fuentes A.J., Tovar S., Pinilla L., Tena-Sempere M., Dieguez C., Castano J.P., Malagon M.M. (2007). Regulation of pituitary cell function by adiponectin. Endocrinology.

[115] Saarela T., Hiltunen M., Helisalmi S., Heinonen S., Laakso M. (2006). Adiponectin gene haplotype is associated with preeclampsia. Genet. Test..

[116] Carmina E., Orio F., Palomba S., Cascella T., Longo R.A., Colao A.M., Lombardi G., Lobo R.A. (2005). Evidence for altered adipocyte function in polycystic ovary syndrome. Eur. J. Endocrinol..

[117] Toulis K.A., Goulis D.G., Farmakiotis D., Georgopoulos N.A., Katsikis I., Tarlatzis B.C., Papadimas I., Panidis D. (2009). Adiponectin levels in women with polycystic ovary syndrome: A systematic review and a meta-analysis. Hum. Reprod. Update.

[118] Michalakis K.G., Segars J.H. (2010). The role of adiponectin in reproduction: From polycystic ovary syndrome to assisted reproduction. Fertil. Steril..

[119] Zhang Y., Proenca R., Maffei M., Barone M., Leopold L., Friedman J.M. (1994). Positional cloning of the mouse obese gene and its human homologue. Nature.

[120] Considine R.V., Sinha M.K., Heiman M.L., Kriauciunas A., Stephens T.W., Nyce M.R., Ohannesian J.P., Marco C.C., McKee L.J., Bauer T.L. (1996). Serum immunoreactive-leptin concentrations in normal-weight and obese humans. N. Engl. J. Med..

[121] Shimizu H., Shimomura Y., Nakanishi Y., Futawatari T., Ohtani K., Sato N., Mori M. (1997). Estrogen increases *in vivo* leptin production in rats and human subjects. J. Endocrinol..

[122] Luukkaa V., Pesonen U., Huhtaniemi I., Lehtonen A., Tilvis R., Tuomilehto J., Koulu M., Huupponen R. (1998). Inverse correlation between serum testosterone and leptin in men. J. Clin. Endocrinol. Metab..

[123] Ahima R.S., Prabakaran D., Mantzoros C., Qu D., Lowell B., Maratos-Flier E., Flier J.S. (1996). Role of leptin in the neuroendocrine response to fasting. Nature.

[124] Chan J.L., Mantzoros C.S. (2005). Role of leptin in energy-deprivation states: Normal human physiology and clinical implications for hypothalamic amenorrhoea and anorexia nervosa. Lancet.

[125] Moschos S., Chan J.L., Mantzoros C.S. (2002). Leptin and reproduction: A review. Fertil. Steril..

[126] Cunningham M.J., Clifton D.K., Steiner R.A. (1999). Leptin’s actions on the reproductive axis: Perspectives and mechanisms. Biol. Reprod..

[127] Quennell J.H., Mulligan A.C., Tups A., Liu X., Phipps S.J., Kemp C.J., Herbison A.E., Grattan D.R., Anderson G.M. (2009). Leptin indirectly regulates gonadotropin-releasing hormone neuronal function. Endocrinology.

[128] Garcia-Galiano D., Allen S.J., Elias C.F. (2014). Role of the adipocyte-derived hormone leptin in reproductive control. Horm. Mol. Biol. Clin. Investig..

[129] Chehab F.F., Lim M.E., Lu R. (1996). Correction of the sterility defect in homozygous obese female mice by treatment with the human recombinant leptin. Nat. Genet..

[130] Welt C.K., Chan J.L., Bullen J., Murphy R., Smith P., DePaoli A.M., Karalis A., Mantzoros C.S. (2004). Recombinant human leptin in women with hypothalamic amenorrhea. N. Engl. J. Med..

[131] Perez-Perez A., Sanchez-Jimenez F., Maymo J., Duenas J.L., Varone C., Sanchez-Margalet V. (2015). Role of leptin in female reproduction. Clin. Chem. Lab. Med..

[132] Karlsson C., Lindell K., Svensson E., Bergh C., Lind P., Billig H., Carlsson L.M., Carlsson B. (1997). Expression of functional leptin receptors in the human ovary. J. Clin. Endocrinol. Metab..

[133] Cioffi J.A., Van Blerkom J., Antczak M., Shafer A., Wittmer S., Snodgrass H.R. (1997). The expression of leptin and its receptors in pre-ovulatory human follicles. Mol. Hum. Reprod..

[134] Archanco M., Muruzabal F.J., Llopiz D., Garayoa M., Gomez-Ambrosi J., Fruhbeck G., Burrell M.A. (2003). Leptin expression in the rat ovary depends on estrous cycle. J. Histochem. Cytochem..

[135] Spicer L.J., Francisco C.C. (1997). The adipose obese gene product, leptin: Evidence of a direct inhibitory role in ovarian function. Endocrinology.

[136] Ghizzoni L., Barreca A., Mastorakos G., Furlini M., Vottero A., Ferrari B., Chrousos G.P., Bernasconi S. (2001). Leptin inhibits steroid biosynthesis by human granulosa-lutein cells. Horm. Metab. Res..

[137] Kendall N.R., Gutierrez C.G., Scaramuzzi R.J., Baird D.T., Webb R., Campbell B.K. (2004). Direct *in vivo* effects of leptin on ovarian steroidogenesis in sheep. Reproduction.

[138] Duggal P.S., Van Der Hoek K.H., Milner C.R., Ryan N.K., Armstrong D.T., Magoffin D.A., Norman R.J. (2000). The *in vivo* and *in vitro* effects of exogenous leptin on ovulation in the rat. Endocrinology.

[139] Brzechffa P.R., Jakimiuk A.J., Agarwal S.K., Weitsman S.R., Buyalos R.P., Magoffin D.A. (1996). Serum immunoreactive leptin concentrations in women with polycystic ovary syndrome. J. Clin. Endocrinol. Metab..

[140] Vicennati V., Gambineri A., Calzoni F., Casimirri F., Macor C., Vettor R., Pasquali R. (1998). Serum leptin in obese women with polycystic ovary syndrome is correlated with body weight and fat distribution but not with androgen and insulin levels. Metabolism.

[141] El Orabi H., Ghalia A.A., Khalifa A., Mahfouz H., El Shalkani A., Shoieb N. (1999). Serum leptin as an additional possible pathogenic factor in polycystic ovary syndrome. Clin. Biochem..

[142] Brannian J.D., Hansen K.A. (2002). Leptin and ovarian folliculogenesis: Implications for ovulation induction and ART outcomes. Semin. Reprod. Med..

[143] Pehlivanov B., Mitkov M. (2009). Serum leptin levels correlate with clinical and biochemical indices of insulin resistance in women with polycystic ovary syndrome. Eur. J. Contracept. Reprod. Health Care.

[144] Yildizhan R., Ilhan G.A., Yildizhan B., Kolusari A., Adali E., Bugdayci G. (2011). Serum retinol-binding protein 4, leptin, and plasma asymmetric dimethylarginine levels in obese and nonobese young women with polycystic ovary syndrome. Fertil. Steril..

[145] Wang L., Li S., Zhao A., Tao T., Mao X., Zhang P., Liu W. (2012). The expression of sex steroid synthesis and inactivation enzymes in subcutaneous adipose tissue of PCOS patients. J. Steroid Biochem. Mol. Biol..

[146] Rizk N.M., Sharif E. (2015). Leptin as well as Free Leptin Receptor Is Associated with Polycystic Ovary Syndrome in Young Women. Int. J. Endocrinol..

[147] Chapman I.M., Wittert G.A., Norman R.J. (1997). Circulating leptin concentrations in polycystic ovary syndrome: Relation to anthropometric and metabolic parameters. Clin. Endocrinol..

[148] Laughlin G.A., Morales A.J., Yen S.S. (1997). Serum leptin levels in women with polycystic ovary syndrome: The role of insulin resistance/hyperinsulinemia. J. Clin. Endocrinol. Metab..

[149] Mantzoros C.S., Dunaif A., Flier J.S. (1997). Leptin concentrations in the polycystic ovary syndrome. J. Clin. Endocrinol. Metab..

[150] Micic D., Macut D., Popovic V., Sumarac-Dumanovic M., Kendereski A., Colic M., Dieguez C., Casanueva F.F. (1997). Leptin levels and insulin sensitivity in obese and non-obese patients with polycystic ovary syndrome. Gynecol. Endocrinol..

[151] Rouru J., Anttila L., Koskinen P., Penttila T.A., Irjala K., Huupponen R., Koulu M. (1997). Serum leptin concentrations in women with polycystic ovary syndrome. J. Clin. Endocrinol. Metab..

[152] Gennarelli G., Holte J., Wide L., Berne C., Lithell H. (1998). Is there a role for leptin in the endocrine and metabolic aberrations of polycystic ovary syndrome?. Hum. Reprod..

[153] Carmina E., Bucchieri S., Mansueto P., Rini G., Ferin M., Lobo R.A. (2009). Circulating levels of adipose products and differences in fat distribution in the ovulatory and anovulatory phenotypes of polycystic ovary syndrome. Fertil. Steril..

[154] Woods A., Johnstone S.R., Dickerson K., Leiper F.C., Fryer L.G., Neumann D., Schlattner U., Wallimann T., Carlson M., Carling D. (2003). LKB1 is the upstream kinase in the AMP-activated protein kinase cascade. Curr. Biol..

[155] Hawley S.A., Pan D.A., Mustard K.J., Ross L., Bain J., Edelman A.M., Frenguelli B.G., Hardie D.G. (2005). Calmodulin-dependent protein kinase kinase-beta is an alternative upstream kinase for AMP-activated protein kinase. Cell Metab..

[156] Hardie D.G. (2015). AMPK: Positive and negative regulation, and its role in whole-body energy homeostasis. Curr. Opin. Cell Biol..

[157] Grahame Hardie D. (2014). AMP-activated protein kinase: A key regulator of energy balance with many roles in human disease. J. Intern. Med..

[158] Steinberg G.R., Kemp B.E. (2009). AMPK in Health and Disease. Physiol. Rev..

[159] Kahn B.B., Alquier T., Carling D., Hardie D.G. (2005). AMP-activated protein kinase: Ancient energy gauge provides clues to modern understanding of metabolism. Cell Metab..

[160] Minokoshi Y., Alquier T., Furukawa N., Kim Y.B., Lee A., Xue B., Mu J., Foufelle F., Ferre P., Birnbaum M.J. (2004). AMP-kinase regulates food intake by responding to hormonal and nutrient signals in the hypothalamus. Nature.

[161] Andersson U., Filipsson K., Abbott C.R., Woods A., Smith K., Bloom S.R., Carling D., Small C.J. (2004). AMP-activated protein kinase plays a role in the control of food intake. J. Biol. Chem..

[162] Mountjoy P.D., Bailey S.J., Rutter G.A. (2007). Inhibition by glucose or leptin of hypothalamic neurons expressing neuropeptide Y requires changes in AMP-activated protein kinase activity. Diabetologia.

[163] Kubota N., Yano W., Kubota T., Yamauchi T., Itoh S., Kumagai H., Kozono H., Takamoto I., Okamoto S., Shiuchi T. (2007). Adiponectin stimulates AMP-activated protein kinase in the hypothalamus and increases food intake. Cell Metab..

[164] Hasenour C.M., Berglund E.D., Wasserman D.H. (2013). Emerging role of AMP-activated protein kinase in endocrine control of metabolism in the liver. Mol. Cell. Endocrinol..

[165] Lim C.T., Kola B., Korbonits M. (2010). AMPK as a mediator of hormonal signalling. J. Mol. Endocrinol..

[166] Viollet B., Athea Y., Mounier R., Guigas B., Zarrinpashneh E., Horman S., Lantier L., Hebrard S., Devin-Leclerc J., Beauloye C. (2009). AMPK: Lessons from transgenic and knockout animals. Front. Biosci..

[167] Steinberg G.R., Rush J.W., Dyck D.J. (2003). AMPK expression and phosphorylation are increased in rodent muscle after chronic leptin treatment. Am. J. Physiol. Endocrinol. Metab..

[168] Yu X., McCorkle S., Wang M., Lee Y., Li J., Saha A.K., Unger R.H., Ruderman N.B. (2004). Leptinomimetic effects of the AMP kinase activator AICAR in leptin-resistant rats: Prevention of diabetes and ectopic lipid deposition. Diabetologia.

[169] Yamauchi T., Kamon J., Minokoshi Y., Ito Y., Waki H., Uchida S., Yamashita S., Noda M., Kita S., Ueki K. (2002). Adiponectin stimulates glucose utilization and fatty-acid oxidation by activating AMP-activated protein kinase. Nat. Med..

[170] Yamauchi T., Nio Y., Maki T., Kobayashi M., Takazawa T., Iwabu M., Okada-Iwabu M., Kawamoto S., Kubota N., Kubota T. (2007). Targeted disruption of AdipoR1 and AdipoR2 causes abrogation of adiponectin binding and metabolic actions. Nat. Med..

[171] Tosca L., Froment P., Solnais P., Ferre P., Foufelle F., Dupont J. (2005). Adenosine 5′-monophosphate-activated protein kinase regulates progesterone secretion in rat granulosa cells. Endocrinology.

[172] Dupont J., Reverchon M., Cloix L., Froment P., Rame C. (2012). Involvement of adipokines, AMPK, PI3K and the PPAR signaling pathways in ovarian follicle development and cancer. Int. J. Dev. Biol..

[173] Tartarin P., Guibert E., Toure A., Ouiste C., Leclerc J., Sanz N., Briere S., Dacheux J.L., Delaleu B., McNeilly J.R. (2012). Inactivation of AMPKalpha1 induces asthenozoospermia and alters spermatozoa morphology. Endocrinology.

[174] Tosca L., Crochet S., Ferre P., Foufelle F., Tesseraud S., Dupont J. (2006). AMP-activated protein kinase activation modulates progesterone secretion in granulosa cells from hen preovulatory follicles. J. Endocrinol..

[175] Nguyen T.M., Alves S., Grasseau I., Metayer-Coustard S., Praud C., Froment P., Blesbois E. (2014). Central role of 5′-AMP-activated protein kinase in chicken sperm functions. Biol. Reprod..

[176] Tosca L., Chabrolle C., Uzbekova S., Dupont J. (2007). Effects of metformin on bovine granulosa cells steroidogenesis: Possible involvement of adenosine 5′ monophosphate-activated protein kinase (AMPK). Biol. Reprod..

[177] Mayes M.A., Laforest M.F., Guillemette C., Gilchrist R.B., Richard F.J. (2007). Adenosine 5′-monophosphate kinase-activated protein kinase (PRKA) activators delay meiotic resumption in porcine oocytes. Biol. Reprod..

[178] Downs S.M., Ya R., Davis C.C. (2010). Role of AMPK throughout meiotic maturation in the mouse oocyte: Evidence for promotion of polar body formation and suppression of premature activation. Mol. Reprod. Dev..

[179] Pellatt L.J., Rice S., Mason H.D. (2011). Phosphorylation and activation of AMP-activated protein kinase (AMPK) by metformin in the human ovary requires insulin. Endocrinology.

[180] Bertoldo M.J., Faure M., Dupont J., Froment P. (2015). AMPK: A master energy regulator for gonadal function. Front. Neurosci..

[181] Tosca L., Solnais P., Ferre P., Foufelle F., Dupont J. (2006). Metformin-induced stimulation of adenosine 5′ monophosphate-activated protein kinase (PRKA) impairs progesterone secretion in rat granulosa cells. Biol. Reprod..

[182] la Marca A., Egbe T.O., Morgante G., Paglia T., Cianci A., De Leo V. (2000). Metformin treatment reduces ovarian cytochrome P-450c17alpha response to human chorionic gonadotrophin in women with insulin resistance-related polycystic ovary syndrome. Hum. Reprod..

[183] Bunger M., Hooiveld G.J., Kersten S., Muller M. (2007). Exploration of PPAR functions by microarray technology--a paradigm for nutrigenomics. Biochim. Biophys. Acta.

[184] Forman B.M., Chen J., Evans R.M. (1997). Hypolipidemic drugs, polyunsaturated fatty acids, and eicosanoids are ligands for peroxisome proliferator-activated receptors alpha and delta. Proc. Natl. Acad. Sci. USA.

[185] Sanderson L.M., de Groot P.J., Hooiveld G.J., Koppen A., Kalkhoven E., Muller M., Kersten S. (2008). Effect of synthetic dietary triglycerides: A novel research paradigm for nutrigenomics. PLoS ONE.

[186] Keller H., Dreyer C., Medin J., Mahfoudi A., Ozato K., Wahli W. (1993). Fatty acids and retinoids control lipid metabolism through activation of peroxisome proliferator-activated receptor-retinoid X receptor heterodimers. Proc. Natl. Acad. Sci. USA.

[187] Mitro N., Mak P.A., Vargas L., Godio C., Hampton E., Molteni V., Kreusch A., Saez E. (2007). The nuclear receptor LXR is a glucose sensor. Nature.

[188] Ide T., Shimano H., Yoshikawa T., Yahagi N., Amemiya-Kudo M., Matsuzaka T., Nakakuki M., Yatoh S., Iizuka Y., Tomita S. (2003). Cross-talk between peroxisome proliferator-activated receptor (PPAR) alpha and liver X receptor (LXR) in nutritional regulation of fatty acid metabolism. II. LXRs suppress lipid degradation gene promoters through inhibition of PPAR signaling. Mol. Endocrinol..

[189] Calkin A.C., Tontonoz P. (2012). Transcriptional integration of metabolism by the nuclear sterol-activated receptors LXR and FXR. Nat. Rev. Mol. Cell. Biol..

[190] Beltowski J., Semczuk A. (2010). Liver X receptor (LXR) and the reproductive system--a potential novel target for therapeutic intervention. Pharmacol. Rep..

[191] Froment P., Gizard F., Defever D., Staels B., Dupont J., Monget P. (2006). Peroxisome proliferator-activated receptors in reproductive tissues: From gametogenesis to parturition. J. Endocrinol..

[192] Froment P. (2008). PPARs and RXRs in Male and Female Fertility and Reproduction. PPAR Res..

[193] Lobaccaro J.M., Gallot D., Lumbroso S., Mouzat K. (2013). Liver X Receptors and female reproduction: When cholesterol meets fertility!. J. Endocrinol. Investig..

[194] Minge C.E., Robker R.L., Norman R.J. (2008). PPAR Gamma: Coordinating Metabolic and Immune Contributions to Female Fertility. PPAR Res..

[195] Steffensen K.R., Robertson K., Gustafsson J.A., Andersen C.Y. (2006). Reduced fertility and inability of oocytes to resume meiosis in mice deficient of the Lxr genes. Mol. Cell. Endocrinol..

[196] Velez L.M., Abruzzese G.A., Motta A.B. (2013). The biology of the peroxisome proliferator-activated receptor system in the female reproductive tract. Curr. Pharm. Des..

[197] Martin J.A., Hamilton B.E., Sutton P.D., Ventura S.J., Mathews T.J., Kirmeyer S., Osterman M.J. (2010). Births: Final data for 2007. Natl. Vital Stat. Rep..

[198] Soules M.R., Bremner W.J. (1982). The menopause and climacteric: Endocrinologic basis and associated symptomatology. J. Am. Geriatr. Soc..

[199] Schwartz D., Mayaux M.J. (1982). Female fecundity as a function of age: Results of artificial insemination in 2193 nulliparous women with azoospermic husbands. Federation CECOS. N. Engl. J. Med..

[200] Eaton S.B., Konner M. (1985). Paleolithic nutrition. A consideration of its nature and current implications. N. Engl. J. Med..

[201] Simopoulos A.P. (2011). Importance of the omega-6/omega-3 balance in health and disease: Evolutionary aspects of diet. World Rev. Nutr. Diet..

[202] Bialostosky K., Wright J.D., Kennedy-Stephenson J., McDowell M., Johnson C.L. (2002). Dietary intake of macronutrients, micronutrients, and other dietary constituents: United States 1988–94. Vital Health Stat..

[203] Hulshof K.F., van Erp-Baart M.A., Anttolainen M., Becker W., Church S.M., Couet C., Hermann-Kunz E., Kesteloot H., Leth T., Martins I. (1999). Intake of fatty acids in western Europe with emphasis on trans fatty acids: The TRANSFAIR Study. Eur. J. Clin. Nutr..

[204] Connor W.E. (2000). Importance of *n*-3 fatty acids in health and disease. Am. J. Clin. Nutr..

[205] Hornstra G. (2000). Essential fatty acids in mothers and their neonates. Am. J. Clin. Nutr..

[206] Huffman S.L., Harika R.K., Eilander A., Osendarp S.J. (2011). Essential fats: How do they affect growth and development of infants and young children in developing countries? A literature review. Matern. Child Nutr..

[207] Bjermo H., Iggman D., Kullberg J., Dahlman I., Johansson L., Persson L., Berglund J., Pulkki K., Basu S., Uusitupa M. (2012). Effects of *n*-6 PUFAs compared with SFAs on liver fat, lipoproteins, and inflammation in abdominal obesity: A randomized controlled trial. Am. J. Clin. Nutr..

[208] Chiuve S.E., Rimm E.B., Sandhu R.K., Bernstein A.M., Rexrode K.M., Manson J.E., Willett W.C., Albert C.M. (2012). Dietary fat quality and risk of sudden cardiac death in women. Am. J. Clin. Nutr..

[209] Livingstone K.M., Lovegrove J.A., Givens D.I. (2012). The impact of substituting SFA in dairy products with MUFA or PUFA on CVD risk: Evidence from human intervention studies. Nutr. Res. Rev..

[210] Michas G., Micha R., Zampelas A. (2014). Dietary fats and cardiovascular disease: Putting together the pieces of a complicated puzzle. Atherosclerosis.

[211] Senthil Kumar S.P., Shen M., Spicer E.G., Goudjo-Ako A.J., Stumph J.D., Zhang J., Shi H. (2014). Distinct metabolic effects following short-term exposure of different high-fat diets in male and female mice. Endocr. J..

[212] Simopoulos A.P. (2008). The importance of the omega-6/omega-3 fatty acid ratio in cardiovascular disease and other chronic diseases. Exp. Biol. Med..

[213] Krishnan S., Cooper J.A. (2014). Effect of dietary fatty acid composition on substrate utilization and body weight maintenance in humans. Eur. J. Nutr..

[214] Berger J., Moller D.E. (2002). The mechanisms of action of PPARs. Annu. Rev. Med..

[215] Brown J.M., Boysen M.S., Jensen S.S., Morrison R.F., Storkson J., Lea-Currie R., Pariza M., Mandrup S., McIntosh M.K. (2003). Isomer-specific regulation of metabolism and PPARgamma signaling by CLA in human preadipocytes. J. Lipid Res..

[216] Saravanan N., Haseeb A., Ehtesham N.Z. (2005). Ghafoorunissa: Differential effects of dietary saturated and trans-fatty acids on expression of genes associated with insulin sensitivity in rat adipose tissue. Eur. J. Endocrinol..

[217] Lefevre M., Lovejoy J.C., Smith S.R., Delany J.P., Champagne C., Most M.M., Denkins Y., de Jonge L., Rood J., Bray G.A. (2005). Comparison of the acute response to meals enriched with cis- or trans-fatty acids on glucose and lipids in overweight individuals with differing FABP2 genotypes. Metabolism.

[218] Salmeron J., Hu F.B., Manson J.E., Stampfer M.J., Colditz G.A., Rimm E.B., Willett W.C. (2001). Dietary fat intake and risk of type 2 diabetes in women. Am. J. Clin. Nutr..

[219] Baer D.J., Judd J.T., Clevidence B.A., Tracy R.P. (2004). Dietary fatty acids affect plasma markers of inflammation in healthy men fed controlled diets: A randomized crossover study. Am. J. Clin. Nutr..

[220] Mozaffarian D., Pischon T., Hankinson S.E., Rifai N., Joshipura K., Willett W.C., Rimm E.B. (2004). Dietary intake of trans fatty acids and systemic inflammation in women. Am. J. Clin. Nutr..

[221] Kasim-Karakas S.E., Almario R.U., Gregory L., Wong R., Todd H., Lasley B.L. (2004). Metabolic and endocrine effects of a polyunsaturated fatty acid-rich diet in polycystic ovary syndrome. J. Clin. Endocrinol. Metab..

[222] O’Connor A., Gibney J., Roche H.M. (2010). Metabolic and hormonal aspects of polycystic ovary syndrome: The impact of diet. Proc. Nutr. Soc..

[223] Phelan N., O’Connor A., Kyaw Tun T., Correia N., Boran G., Roche H.M., Gibney J. (2011). Hormonal and metabolic effects of polyunsaturated fatty acids in young women with polycystic ovary syndrome: Results from a cross-sectional analysis and a randomized, placebo-controlled, crossover trial. Am. J. Clin. Nutr..

[224] Mohammadi E., Rafraf M., Farzadi L., Asghari-Jafarabadi M., Sabour S. (2012). Effects of omega-3 fatty acids supplementation on serum adiponectin levels and some metabolic risk factors in women with polycystic ovary syndrome. Asia Pac. J. Clin. Nutr..

[225] Liepa G.U., Sengupta A., Karsies D. (2008). Polycystic ovary syndrome (PCOS) and other androgen excess-related conditions: Can changes in dietary intake make a difference?. Nutr. Clin. Pract..

[226] Kalgaonkar S., Almario R.U., Gurusinghe D., Garamendi E.M., Buchan W., Kim K., Karakas S.E. (2011). Differential effects of walnuts vs almonds on improving metabolic and endocrine parameters in PCOS. Eur. J. Clin. Nutr..

[227] Kris-Etherton P.M., Binkoski A.E., Zhao G., Coval S.M., Clemmer K.F., Hecker K.D., Jacques H., Etherton T.D. (2002). Dietary fat: Assessing the evidence in support of a moderate-fat diet; the benchmark based on lipoprotein metabolism. Proc. Nutr. Soc..

[228] Baird D.T., Collins J., Egozcue J., Evers L.H., Gianaroli L., Leridon H., Sunde A., Templeton A., Van Steirteghem A., Cohen J. (2005). Fertility and ageing. Hum. Reprod. Update.

[229] Abayasekara D.R., Wathes D.C. (1999). Effects of altering dietary fatty acid composition on prostaglandin synthesis and fertility. Prostaglandins Leukot. Essent. Fat. Acids.

[230] Coyne G.S., Kenny D.A., Childs S., Sreenan J.M., Waters S.M. (2008). Dietary *n*-3 polyunsaturated fatty acids alter the expression of genes involved in prostaglandin biosynthesis in the bovine uterus. Theriogenology.

[231] Mattos R., Staples C.R., Thatcher W.W. (2000). Effects of dietary fatty acids on reproduction in ruminants. Rev. Reprod..

[232] Robinson R.S., Pushpakumara P.G., Cheng Z., Peters A.R., Abayasekara D.R., Wathes D.C. (2002). Effects of dietary polyunsaturated fatty acids on ovarian and uterine function in lactating dairy cows. Reproduction.

[233] Marei W.F., Wathes D.C., Fouladi-Nashta A.A. (2010). Impact of linoleic acid on bovine oocyte maturation and embryo development. Reproduction.

[234] Yoshikawa T., Shimano H., Yahagi N., Ide T., Amemiya-Kudo M., Matsuzaka T., Nakakuki M., Tomita S., Okazaki H., Tamura Y. (2002). Polyunsaturated fatty acids suppress sterol regulatory element-binding protein 1c promoter activity by inhibition of liver X receptor (LXR) binding to LXR response elements. J. Biol. Chem..

[235] Komar C.M. (2005). Peroxisome proliferator-activated receptors (PPARs) and ovarian function—Implications for regulating steroidogenesis, differentiation, and tissue remodeling. Reprod. Biol. Endocrinol..

[236] Sampath H., Ntambi J.M. (2005). Polyunsaturated fatty acid regulation of genes of lipid metabolism. Annu. Rev. Nutr..

[237] Hughes J., Kwong W.Y., Li D., Salter A.M., Lea R.G., Sinclair K.D. (2011). Effects of omega-3 and -6 polyunsaturated fatty acids on ovine follicular cell steroidogenesis, embryo development and molecular markers of fatty acid metabolism. Reproduction.

[238] Wonnacott K.E., Kwong W.Y., Hughes J., Salter A.M., Lea R.G., Garnsworthy P.C., Sinclair K.D. (2010). Dietary omega-3 and -6 polyunsaturated fatty acids affect the composition and development of sheep granulosa cells, oocytes and embryos. Reproduction.

[239] Kim P.Y., Zhong M., Kim Y.S., Sanborn B.M., Allen K.G. (2012). Long chain polyunsaturated fatty acids alter oxytocin signaling and receptor density in cultured pregnant human myometrial smooth muscle cells. PLoS ONE.

[240] Wathes D.C., Abayasekara D.R., Aitken R.J. (2007). Polyunsaturated fatty acids in male and female reproduction. Biol. Reprod..

[241] Oken E., Kleinman K.P., Olsen S.F., Rich-Edwards J.W., Gillman M.W. (2004). Associations of seafood and elongated *n*-3 fatty acid intake with fetal growth and length of gestation: Results from a US pregnancy cohort. Am. J. Epidemiol..

[242] Horvath A., Koletzko B., Szajewska H. (2007). Effect of supplementation of women in high-risk pregnancies with long-chain polyunsaturated fatty acids on pregnancy outcomes and growth measures at birth: A meta-analysis of randomized controlled trials. Br. J. Nutr..

[243] Nehra D., Le H.D., Fallon E.M., Carlson S.J., Woods D., White Y.A., Pan A.H., Guo L., Rodig S.J., Tilly J.L. (2012). Prolonging the female reproductive lifespan and improving egg quality with dietary omega-3 fatty acids. Aging Cell.

[244] Olsen S.F., Sorensen J.D., Secher N.J., Hedegaard M., Henriksen T.B., Hansen H.S., Grant A. (1992). Randomised controlled trial of effect of fish-oil supplementation on pregnancy duration. Lancet.

[245] Helland I.B., Saugstad O.D., Smith L., Saarem K., Solvoll K., Ganes T., Drevon C.A. (2001). Similar effects on infants of *n*-3 and *n*-6 fatty acids supplementation to pregnant and lactating women. Pediatrics.

[246] Szajewska H., Horvath A., Koletzko B. (2006). Effect of *n*-3 long-chain polyunsaturated fatty acid supplementation of women with low-risk pregnancies on pregnancy outcomes and growth measures at birth: A meta-analysis of randomized controlled trials. Am. J. Clin. Nutr..

[247] Zachut M., Arieli A., Moallem U. (2011). Incorporation of dietary *n*-3 fatty acids into ovarian compartments in dairy cows and the effects on hormonal and behavioral patterns around estrus. Reproduction.

[248] Lammoglia M.A., Willard S.T., Hallford D.M., Randel R.D. (1997). Effects of dietary fat on follicular development and circulating concentrations of lipids, insulin, progesterone, estradiol-17 beta, 13,14-dihydro-15-keto-prostaglandin F(2 alpha), and growth hormone in estrous cyclic Brahman cows. J. Anim. Sci..

[249] Grummer R.R., Carroll D.J. (1991). Effects of dietary fat on metabolic disorders and reproductive performance of dairy cattle. J. Anim. Sci..

[250] Staples C.R., Burke J.M., Thatcher W.W. (1998). Influence of supplemental fats on reproductive tissues and performance of lactating cows. J. Dairy Sci..

[251] Sangsritavong S., Combs D.K., Sartori R., Armentano L.E., Wiltbank M.C. (2002). High feed intake increases liver blood flow and metabolism of progesterone and estradiol-17beta in dairy cattle. J. Dairy Sci..

[252] Wiltbank M., Lopez H., Sartori R., Sangsritavong S., Gumen A. (2006). Changes in reproductive physiology of lactating dairy cows due to elevated steroid metabolism. Theriogenology.

[253] Piccinato C.A., Sartori R., Sangsritavong S., Souza A.H., Grummer R.R., Luchini D., Wiltbank M.C. (2010). *In vitro* and *in vivo* analysis of fatty acid effects on metabolism of 17beta-estradiol and progesterone in dairy cows. J. Dairy Sci..

[254] Hirunpanich V., Katagi J., Sethabouppha B., Sato H. (2006). Demonstration of docosahexaenoic acid as a bioavailability enhancer for CYP3A substrates: *In vitro* and *in vivo* evidence using cyclosporin in rats. Drug Metab. Dispos..

[255] Yao H.T., Chang Y.W., Lan S.J., Chen C.T., Hsu J.T., Yeh T.K. (2006). The inhibitory effect of polyunsaturated fatty acids on human CYP enzymes. Life Sci..

[256] Glidewell-Kenney C., Hurley L.A., Pfaff L., Weiss J., Levine J.E., Jameson J.L. (2007). Nonclassical estrogen receptor alpha signaling mediates negative feedback in the female mouse reproductive axis. Proc. Natl. Acad. Sci. USA.

[257] Ojeda S.R., Negro-Vilar A., McCann S.M. (1979). Release of prostaglandin Es by hypothalamic tissue: Evidence for their involvement in catecholamine-induced luteinizing hormone-releasing hormone release. Endocrinology.

[258] Kim K., Ramirez V.D. (1986). Effects of prostaglandin E2, forskolin and cholera toxin on cAMP production and *in vitro* LH-RH release from the rat hypothalamus. Brain Res..

[259] McNatty K.P., Makris A., DeGrazia C., Osathanondh R., Ryan K.J. (1979). The production of progesterone, androgens, and estrogens by granulosa cells, thecal tissue, and stromal tissue from human ovaries *in vitro*. J. Clin. Endocrinol. Metab..

[260] Ireland J.J., Roche J.F. (1982). Development of antral follicles in cattle after prostaglandin-induced luteolysis: Changes in serum hormones, steroids in follicular fluid, and gonadotropin receptors. Endocrinology.

[261] Zachut M., Arieli A., Lehrer H., Argov N., Moallem U. (2008). Dietary unsaturated fatty acids influence preovulatory follicle characteristics in dairy cows. Reproduction.

[262] Ouladsahebmadarek E., Khaki A., Khanahmadi S., Ahmadi Ashtiani H., Paknejad P., Ayubi M.R. (2014). Hormonal and metabolic effects of polyunsaturated fatty acid (omega-3) on polycystic ovary syndrome induced rats under diet. Iran. J. Basic Med. Sci..

[263] Douglas C.C., Gower B.A., Darnell B.E., Ovalle F., Oster R.A., Azziz R. (2006). Role of diet in the treatment of polycystic ovary syndrome. Fertil. Steril..

[264] Chavarro J.E., Rich-Edwards J.W., Rosner B.A., Willett W.C. (2009). A prospective study of dietary carbohydrate quantity and quality in relation to risk of ovulatory infertility. Eur. J. Clin. Nutr..

[265] Kaaks R., Lukanova A. (2001). Energy balance and cancer: The role of insulin and insulin-like growth factor-I. Proc. Nutr. Soc..

[266] Boyd N.F., Lockwood G.A., Greenberg C.V., Martin L.J., Tritchler D.L. (1997). Effects of a low-fat high-carbohydrate diet on plasma sex hormones in premenopausal women: Results from a randomized controlled trial. Canadian Diet and Breast Cancer Prevention Study Group. Br. J. Cancer.

[267] Sherman B.M., West J.H., Korenman S.G. (1976). The menopausal transition: Analysis of, L.H.; FSH, estradiol, and progesterone concentrations during menstrual cycles of older women. J. Clin. Endocrinol. Metab..

[268] Cui X., Rosner B., Willett W.C., Hankinson S.E. (2010). Dietary fat, fiber, and carbohydrate intake and endogenous hormone levels in premenopausal women. Horm. Cancer.

[269] Boyd N.F., Greenberg C., Martin L., Stone J., Hammond G., Minkin S. (2002). Lack of effect of a low-fat high-carbohydrate diet on ovarian hormones in premenopausal women: Results from a randomized trial. IARC Sci. Publ..

[270] Chavarro J.E., Rich-Edwards J.W., Rosner B., Willett W.C. (2007). A prospective study of dairy foods intake and anovulatory infertility. Hum. Reprod..

[271] Welsh J.A., Sharma A., Cunningham S.A., Vos M.B. (2011). Consumption of added sugars and indicators of cardiovascular disease risk among US adolescents. Circulation.

[272] Lucero J., Harlow B.L., Barbieri R.L., Sluss P., Cramer D.W. (2001). Early follicular phase hormone levels in relation to patterns of alcohol, tobacco, and coffee use. Fertil. Steril..

[273] Nagata C., Kabuto M., Shimizu H. (1998). Association of coffee, green tea, and caffeine intakes with serum concentrations of estradiol and sex hormone-binding globulin in premenopausal Japanese women. Nutr. Cancer.

[274] Chavarro J.E., Rich-Edwards J.W., Rosner B.A., Willett W.C. (2009). Caffeinated and alcoholic beverage intake in relation to ovulatory disorder infertility. Epidemiology.

[275] Schliep K.C., Schisterman E.F., Mumford S.L., Pollack A.Z., Perkins N.J., Ye A., Zhang C.J., Stanford J.B., Porucznik C.A., Hammoud A.O. (2013). Energy-containing beverages: Reproductive hormones and ovarian function in the BioCycle Study. Am. J. Clin Nutr..

[276] Stamets K., Taylor D.S., Kunselman A., Demers L.M., Pelkman C.L., Legro R.S. (2004). A randomized trial of the effects of two types of short-term hypocaloric diets on weight loss in women with polycystic ovary syndrome. Fertil. Steril..

[277] Moran L.J., Noakes M., Clifton P.M., Tomlinson L., Galletly C., Norman R.J. (2003). Dietary composition in restoring reproductive and metabolic physiology in overweight women with polycystic ovary syndrome. J. Clin. Endocrinol. Metab..

[278] Chavarro J.E., Rich-Edwards J.W., Rosner B.A., Willett W.C. (2008). Protein intake and ovulatory infertility. Am. J. Obstet. Gynecol..

[279] Gannon M.C., Nuttall F.Q., Neil B.J., Westphal S.A. (1988). The insulin and glucose responses to meals of glucose plus various proteins in type II diabetic subjects. Metabolism.

[280] Hubbard R., Kosch C.L., Sanchez A., Sabate J., Berk L., Shavlik G. (1989). Effect of dietary protein on serum insulin and glucagon levels in hyper- and normocholesterolemic men. Atherosclerosis.

[281] Holmes M.D., Pollak M.N., Willett W.C., Hankinson S.E. (2002). Dietary correlates of plasma insulin-like growth factor I and insulin-like growth factor binding protein 3 concentrations. Cancer Epidemiol. Biomark. Prev..

[282] Bakan R., Birmingham C.L., Aeberhardt L., Goldner E.M. (1993). Dietary zinc intake of vegetarian and nonvegetarian patients with anorexia nervosa. Int. J. Eat. Disord..

[283] Brooks S.M., Sanborn C.F., Albrecht B.H., Wagner W.W. (1984). Diet in athletic amenorrhoea. Lancet.

[284] Lloyd T., Schaeffer J.M., Walker M.A., Demers L.M. (1991). Urinary hormonal concentrations and spinal bone densities of premenopausal vegetarian and nonvegetarian women. Am. J. Clin. Nutr..

[285] Pedersen A.B., Bartholomew M.J., Dolence L.A., Aljadir L.P., Netteburg K.L., Lloyd T. (1991). Menstrual differences due to vegetarian and nonvegetarian diets. Am. J. Clin. Nutr..

[286] Pirke K.M., Schweiger U., Laessle R., Dickhaut B., Schweiger M., Waechtler M. (1986). Dieting influences the menstrual cycle: Vegetarian *versus* nonvegetarian diet. Fertil. Steril..

[287] Slavin J., Lutter J., Cushman S. (1984). Amenorrhoea in vegetarian athletes. Lancet.

[288] Barr S.I., Janelle K.C., Prior J.C. (1994). Vegetarian vs nonvegetarian diets, dietary restraint, and subclinical ovulatory disturbances: Prospective 6-mo study. Am. J. Clin. Nutr..

[289] Dwyer J.T. (1988). Health aspects of vegetarian diets. Am. J. Clin. Nutr..

[290] Slattery M.L., Jacobs D.R., Caan B.J., Van Horn L., Bragg C., Manolio T.A., Kushi L.H., Liu K.A. (1991). Meat consumption and its associations with other diet and health factors in young adults: The CARDIA study. Am. J. Clin. Nutr..

[291] Barr S.I. (1999). Vegetarianism and menstrual cycle disturbances: Is there an association?. Am. J. Clin. Nutr..

[292] Campbell-Brown M., Ward R.J., Haines A.P., North W.R., Abraham R., McFadyen I.R., Turnlund J.R., King J.C. (1985). Zinc and copper in Asian pregnancies--is there evidence for a nutritional deficiency?. Br. J. Obstet. Gynaecol..

[293] Fonnebo V. (1994). The healthy Seventh-Day Adventist lifestyle: What is the Norwegian experience?. Am. J. Clin. Nutr..

[294] Reddy S., Sanders T.A., Obeid O. (1994). The influence of maternal vegetarian diet on essential fatty acid status of the newborn. Eur. J. Clin. Nutr..

[295] Stuebe A.M., Oken E., Gillman M.W. (2009). Associations of diet and physical activity during pregnancy with risk for excessive gestational weight gain. Am. J. Obstet. Gynecol..

[296] Thomas J., Ellis F.R. (1977). The health of vegans during pregnancy. Proc. Nutr. Soc..

[297] Lakin V., Haggarty P., Abramovich D.R., Ashton J., Moffat C.F., McNeill G., Danielian P.J., Grubb D. (1998). Dietary intake and tissue concentration of fatty acids in omnivore, vegetarian and diabetic pregnancy. Prostaglandins Leukot. Essent. Fat. Acids.

[298] Stammers J.P., Hull D., Abraham R., McFadyen I.R. (1989). High arachidonic acid levels in the cord blood of infants of mothers on vegetarian diets. Br. J. Nutr..

[299] Ward R.J., Abraham R., McFadyen I.R., Haines A.D., North W.R., Patel M., Bhatt R.V. (1988). Assessment of trace metal intake and status in a Gujerati pregnant Asian population and their influence on the outcome of pregnancy. Br. J. Obstet. Gynaecol..

[300] Kane K.K., Creighton K.W., Petersen M.K., Hallford D.M., Remmenga M.D., Hawkins D.E. (2002). Effects of varying levels of undegradable intake protein on endocrine and metabolic function of young post-partum beef cows. Theriogenology.

[301] Quesnel H., Mejia-Guadarrama C.A., Pasquier A., Dourmad J.Y., Prunier A. (2005). Dietary protein restriction during lactation in primiparous sows with different live weights at farrowing: II. Consequences on reproductive performance and interactions with metabolic status. Reprod. Nutr. Dev..

[302] Polkowska J., Przekop F. (1993). Effect of protein deficiency on luteinizing hormone releasing hormone (LHRH), gonadotropin releasing hormone associated peptide (GAP) and luteinizing hormone (LH) immunocytochemistry in the hypothalamus and pituitary gland of prepubertal ewes. Exp. Clin. Endocrinol..

[303] Meza-Herrera C.A., Hallford D.M., Ortiz J.A., Cuevas R.A., Sanchez J.M., Salinas H., Mellado M., Gonzalez-Bulnes A. (2008). Body condition and protein supplementation positively affect periovulatory ovarian activity by non LH-mediated pathways in goats. Anim. Reprod. Sci..

[304] Armstrong D.G., McEvoy T.G., Baxter G., Robinson J.J., Hogg C.O., Woad K.J., Webb R., Sinclair K.D. (2001). Effect of dietary energy and protein on bovine follicular dynamics and embryo production *in vitro*: Associations with the ovarian insulin-like growth factor system. Biol. Reprod..

[305] Jordan E.R., Swanson L.V. (1979). Serum progesterone and luteinizing hormone in dairy cattle fed varying levels of crude protein. J. Anim. Sci..

[306] Sonderman J.P., Larson L.L. (1989). Effect of dietary protein and exogenous gonadotropin-releasing hormone on circulating progesterone concentrations and performance of Holstein cows. J. Dairy Sci..

[307] Barton B.A., Rosario H.A., Anderson G.W., Grindle B.P., Carroll D.J. (1996). Effects of dietary crude protein, breed, parity, and health status on the fertility of dairy cows. J. Dairy Sci..

[308] Blauwiekel R., Kincaid R.L., Reeves J.J. (1986). Effect of high crude protein on pituitary and ovarian function in Holstein cows. J. Dairy Sci..

[309] Garcia-Bojalil C.M., Staples C.R., Thatcher W.W., Drost M. (1994). Protein intake and development of ovarian follicles and embryos of superovulated nonlactating dairy cows. J. Dairy Sci..

[310] Elrod C.C., Butler W.R. (1993). Reduction of fertility and alteration of uterine pH in heifers fed excess ruminally degradable protein. J. Anim. Sci..

[311] Cappellozza B.I., Cooke R.F., Reis M.M., Marques R.S., Guarnieri Filho T.A., Perry G.A., Jump D.B., Lytle K.A., Bohnert D.W. (2015). Effects of protein supplementation frequency on physiological responses associated with reproduction in beef cows. J. Anim. Sci..

[312] Kriengsinyos W., Wykes L.J., Goonewardene L.A., Ball R.O., Pencharz P.B. (2004). Phase of menstrual cycle affects lysine requirement in healthy women. Am. J. Physiol. Endocrinol. Metab..

[313] Lariviere F., Moussalli R., Garrel D.R. (1994). Increased leucine flux and leucine oxidation during the luteal phase of the menstrual cycle in women. Am. J. Physiol..

[314] Wallace M., Hashim Y.Z., Wingfield M., Culliton M., McAuliffe F., Gibney M.J., Brennan L. (2010). Effects of menstrual cycle phase on metabolomic profiles in premenopausal women. Hum. Reprod..

[315] Landau R.L., Lugibihl K. (1961). The effect of progesterone on amino acid metabolism. J. Clin. Endocrinol. Metab..

[316] Moller S.E., Moller B.M., Olesen M., Fjalland B. (1996). Effects of oral contraceptives on plasma neutral amino acids and cholesterol during a menstrual cycle. Eur. J. Clin. Pharmacol.

[317] Demers L.M., Feil P.D., Bardin C.W. (1977). Factors that influence steroid induction of endometrial glycogenesis in organ culture. Ann. N. Y. Acad. Sci..

[318] Shapiro S.S., Dyer S.D., Colas A.E. (1980). Progesterone-induced glycogen accumulation in human endometrium during organ culture. Am. J. Obstet. Gynecol..

[319] Savouret J.F., Misrahi M., Milgrom E. (1990). Molecular action of progesterone. Int. J. Biochem..

[320] Konner M., Eaton S.B. (2010). Paleolithic nutrition: Twenty-five years later. Nutr. Clin. Pract..

[321] Jefferson W.N., Padilla-Banks E., Newbold R.R. (2006). Studies of the effects of neonatal exposure to genistein on the developing female reproductive system. J. AOAC Int..

[322] Fielden M.R., Samy S.M., Chou K.C., Zacharewski T.R. (2003). Effect of human dietary exposure levels of genistein during gestation and lactation on long-term reproductive development and sperm quality in mice. Food Chem. Toxicol..

[323] Sharpe R.M., Fisher J.S., Millar M.M., Jobling S., Sumpter J.P. (1995). Gestational and lactational exposure of rats to xenoestrogens results in reduced testicular size and sperm production. Environ. Health Perspect..

[324] Wang Y., Thuillier R., Culty M. (2004). Prenatal estrogen exposure differentially affects estrogen receptor-associated proteins in rat testis gonocytes. Biol. Reprod..

[325] Diamanti-Kandarakis E., Bourguignon J.P., Giudice L.C., Hauser R., Prins G.S., Soto A.M., Zoeller R.T., Gore A.C. (2009). Endocrine-disrupting chemicals: An Endocrine Society scientific statement. Endocr. Rev..

[326] Fusani L., Della Seta D., Dessi-Fulgheri F., Farabollini F. (2007). Altered reproductive success in rat pairs after environmental-like exposure to xenoestrogen. Proc. Biol Sci..

[327] Maranghi F., Tassinari R., Marcoccia D., Altieri I., Catone T., De Angelis G., Testai E., Mastrangelo S., Evandri M.G., Bolle P. (2010). The food contaminant semicarbazide acts as an endocrine disrupter: Evidence from an integrated *in vivo*/*in vitro* approach. Chem. Biol. Interact..

[328] Matsuura I., Saitoh T., Ashina M., Wako Y., Iwata H., Toyota N., Ishizuka Y., Namiki M., Hoshino N., Tsuchitani M. (2005). Evaluation of a two-generation reproduction toxicity study adding endpoints to detect endocrine disrupting activity using vinclozolin. J. Toxicol. Sci..

[329] Schiffer C., Muller A., Egeberg D.L., Alvarez L., Brenker C., Rehfeld A., Frederiksen H., Waschle B., Kaupp U.B., Balbach M. (2014). Direct action of endocrine disrupting chemicals on human sperm. EMBO Rep..

[330] Schug T.T., Janesick A., Blumberg B., Heindel J.J. (2011). Endocrine disrupting chemicals and disease susceptibility. J. Steroid Biochem. Mol. Biol..

[331] Zawatski W., Lee M.M. (2013). Male pubertal development: Are endocrine-disrupting compounds shifting the norms?. J. Endocrinol..

[332] Soucy N.V., Parkinson H.D., Sochaski M.A., Borghoff S.J. (2006). Kinetics of genistein and its conjugated metabolites in pregnant Sprague-Dawley rats following single and repeated genistein administration. Toxicol. Sci..

[333] Penza M., Montani C., Romani A., Vignolini P., Ciana P., Maggi A., Pampaloni B., Caimi L., Di Lorenzo D. (2007). Genistein accumulates in body depots and is mobilized during fasting, reaching estrogenic levels in serum that counter the hormonal actions of estradiol and organochlorines. Toxicol. Sci..

[334] Doerge D.R., Churchwell M.I., Chang H.C., Newbold R.R., Delclos K.B. (2001). Placental transfer of the soy isoflavone genistein following dietary and gavage administration to Sprague Dawley rats. Reprod. Toxicol..

[335] Jansson T., Powell T.L. (2000). Placental nutrient transfer and fetal growth. Nutrition.

[336] Todaka E., Sakurai K., Fukata H., Miyagawa H., Uzuki M., Omori M., Osada H., Ikezuki Y., Tsutsumi O., Iguchi T. (2005). Fetal exposure to phytoestrogens--the difference in phytoestrogen status between mother and fetus. Environ. Res..

[337] Bateman H.L., Patisaul H.B. (2008). Disrupted female reproductive physiology following neonatal exposure to phytoestrogens or estrogen specific ligands is associated with decreased GnRH activation and kisspeptin fiber density in the hypothalamus. Neurotoxicology.

[338] Navarro V.M., Sanchez-Garrido M.A., Castellano J.M., Roa J., Garcia-Galiano D., Pineda R., Aguilar E., Pinilla L., Tena-Sempere M. (2009). Persistent impairment of hypothalamic KiSS-1 system after exposures to estrogenic compounds at critical periods of brain sex differentiation. Endocrinology.

[339] Warita K., Okamoto K., Mutoh K., Hasegawa Y., Yue Z.P., Yokoyama T., Matsumoto Y., Miki T., Takeuchi Y., Kitagawa H. (2008). Activin A and equine chorionic gonadotropin recover reproductive dysfunction induced by neonatal exposure to an estrogenic endocrine disruptor in adult male mice. Biol. Reprod..

[340] Fujimoto V.Y., Kim D., vom Saal F.S., Lamb J.D., Taylor J.A., Bloom M.S. (2011). Serum unconjugated bisphenol A concentrations in women may adversely influence oocyte quality during *in vitro* fertilization. Fertil. Steril..

[341] Jefferson W., Newbold R., Padilla-Banks E., Pepling M. (2006). Neonatal genistein treatment alters ovarian differentiation in the mouse: Inhibition of oocyte nest breakdown and increased oocyte survival. Biol. Reprod..

[342] Juliani C.C., Silva-Zacarin E.C., Santos D.C., Boer P.A. (2008). Effects of atrazine on female Wistar rats: Morphological alterations in ovarian follicles and immunocytochemical labeling of 90 kDa heat shock protein. Micron.

[343] Susiarjo M., Hassold T.J., Freeman E., Hunt P.A. (2007). Bisphenol A exposure in utero disrupts early oogenesis in the mouse. PLoS Genet..

[344] Uzumcu M., Kuhn P.E., Marano J.E., Armenti A.E., Passantino L. (2006). Early postnatal methoxychlor exposure inhibits folliculogenesis and stimulates anti-Mullerian hormone production in the rat ovary. J. Endocrinol..

[345] Valdez K.E., Shi Z., Ting A.Y., Petroff B.K. (2009). Effect of chronic exposure to the aryl hydrocarbon receptor agonist 2,3,7,8-tetrachlorodibenzo-p-dioxin in female rats on ovarian gene expression. Reprod. Toxicol..

[346] Wang W., Craig Z.R., Basavarajappa M.S., Hafner K.S., Flaws J.A. (2012). Mono-(2-ethylhexyl) phthalate induces oxidative stress and inhibits growth of mouse ovarian antral follicles. Biol. Reprod..

[347] Zhuang X.L., Fu Y.C., Xu J.J., Kong X.X., Chen Z.G., Luo L.L. (2010). Effects of genistein on ovarian follicular development and ovarian life span in rats. Fitoterapia.

[348] Caserta D., Maranghi L., Mantovani A., Marci R., Maranghi F., Moscarini M. (2008). Impact of endocrine disruptor chemicals in gynaecology. Hum. Reprod. Update.

[349] Cobellis L., Colacurci N., Trabucco E., Carpentiero C., Grumetto L. (2009). Measurement of bisphenol A and bisphenol B levels in human blood sera from healthy and endometriotic women. Biomed. Chromatogr..

[350] Missmer S.A., Hankinson S.E., Spiegelman D., Barbieri R.L., Michels K.B., Hunter D.J. (2004). In utero exposures and the incidence of endometriosis. Fertil. Steril..

[351] Reddy B.S., Rozati R., Reddy B.V., Raman N.V. (2006). Association of phthalate esters with endometriosis in Indian women. BJOG.

[352] Uzumcu M., Zachow R. (2007). Developmental exposure to environmental endocrine disruptors: Consequences within the ovary and on female reproductive function. Reprod. Toxicol..

[353] Caserta D., Mantovani A., Marci R., Fazi A., Ciardo F., La Rocca C., Maranghi F., Moscarini M. (2011). Environment and women’s reproductive health. Hum. Reprod. Update.

[354] De Coster S., van Larebeke N. (2012). Endocrine-disrupting chemicals: Associated disorders and mechanisms of action. J. Environ. Public Health.

[355] Arase S., Ishii K., Igarashi K., Aisaki K., Yoshio Y., Matsushima A., Shimohigashi Y., Arima K., Kanno J., Sugimura Y. (2011). Endocrine disrupter bisphenol A increases *in situ* estrogen production in the mouse urogenital sinus. Biol. Reprod..

[356] Boukari K., Ciampi M.L., Guiochon-Mantel A., Young J., Lombes M., Meduri G. (2007). Human fetal testis: Source of estrogen and target of estrogen action. Hum. Reprod..

[357] Craig Z.R., Wang W., Flaws J.A. (2011). Endocrine-disrupting chemicals in ovarian function: Effects on steroidogenesis, metabolism and nuclear receptor signaling. Reproduction.

[358] Holloway A.C., Anger D.A., Crankshaw D.J., Wu M., Foster W.G. (2008). Atrazine-induced changes in aromatase activity in estrogen sensitive target tissues. J. Appl. Toxicol..

[359] Phillips K.P., Foster W.G. (2008). Key developments in endocrine disrupter research and human health. J. Toxicol. Environ. Health B Crit. Rev..

[360] Whitehead S.A., Rice S. (2006). Endocrine-disrupting chemicals as modulators of sex steroid synthesis. Best Pract. Res. Clin. Endocrinol. Metab..

[361] Derouiche L., Keller M., Martini M., Duittoz A.H., Pillon D. (2015). Developmental Exposure to Ethinylestradiol Affects Reproductive Physiology, the GnRH Neuroendocrine Network and Behaviors in Female Mouse. Front. Neurosci..

[362] Davis C., Bryan J., Hodgson J., Murphy K. (2015). Definition of the Mediterranean Diet: A Literature Review. Nutrients.

[363] Willett W.C., Sacks F., Trichopoulou A., Drescher G., Ferro-Luzzi A., Helsing E., Trichopoulos D. (1995). Mediterranean diet pyramid: A cultural model for healthy eating. Am. J. Clin. Nutr..

[364] Serra-Majem L., Roman B., Estruch R. (2006). Scientific evidence of interventions using the Mediterranean diet: A systematic review. Nutr. Rev..

[365] Sofi F., Abbate R., Gensini G.F., Casini A. (2010). Accruing evidence on benefits of adherence to the Mediterranean diet on health: An updated systematic review and meta-analysis. Am. J. Clin. Nutr..

[366] Esposito K., Kastorini C.M., Panagiotakos D.B., Giugliano D. (2013). Mediterranean diet and metabolic syndrome: An updated systematic review. Rev. Endocr. Metab. Disord..

[367] Garcia-Fernandez E., Rico-Cabanas L., Rosgaard N., Estruch R., Bach-Faig A. (2014). Mediterranean diet and cardiodiabesity: A review. Nutrients.

[368] Georgoulis M., Kontogianni M.D., Yiannakouris N. (2014). Mediterranean diet and diabetes: Prevention and treatment. Nutrients.

[369] Giugliano D., Esposito K. (2008). Mediterranean diet and metabolic diseases. Curr. Opin. Lipidol..

[370] Koloverou E., Esposito K., Giugliano D., Panagiotakos D. (2014). The effect of Mediterranean diet on the development of type 2 diabetes mellitus: A meta-analysis of 10 prospective studies and 136,846 participants. Metabolism.

[371] Martinez-Gonzalez M.A., de la Fuente-Arrillaga C., Nunez-Cordoba J.M., Basterra-Gortari F.J., Beunza J.J., Vazquez Z., Benito S., Tortosa A., Bes-Rastrollo M. (2008). Adherence to Mediterranean diet and risk of developing diabetes: Prospective cohort study. BMJ.

[372] Panagiotakos D.B., Pitsavos C., Chrysohoou C., Stefanadis C. (2005). The epidemiology of Type 2 diabetes mellitus in Greek adults: The ATTICA study. Diabet. Med..

[373] Eguaras S., Toledo E., Hernandez-Hernandez A., Cervantes S., Martinez-Gonzalez M.A. (2015). Better Adherence to the Mediterranean Diet Could Mitigate the Adverse Consequences of Obesity on Cardiovascular Disease: The SUN Prospective Cohort. Nutrients.

[374] Estruch R., Ros E., Martinez-Gonzalez M.A. (2013). Mediterranean diet for primary prevention of cardiovascular disease. N. Engl. J. Med..

[375] Estruch R., Ros E., Salas-Salvado J., Covas M.I., Corella D., Aros F., Gomez-Gracia E., Ruiz-Gutierrez V., Fiol M., Lapetra J. (2013). Primary prevention of cardiovascular disease with a Mediterranean diet. N. Engl. J. Med..

[376] Gaskins A.J., Rovner A.J., Mumford S.L., Yeung E., Browne R.W., Trevisan M., Perkins N.J., Wactawski-Wende J., Schisterman E.F. (2010). Adherence to a Mediterranean diet and plasma concentrations of lipid peroxidation in premenopausal women. Am. J. Clin. Nutr..

[377] Mattiello A., Chiodini P., Santucci de Magistris M., Krogh V., Grioni S., Fasanelli F., Vineis P., Saieva C., Bendinelli B., Frasca G. (2015). [Dietary habits and cardiovascular disease: The experience of EPIC Italian collaboration]. Epidemiol. Prev..

[378] Stefler D., Malyutina S., Kubinova R., Pajak A., Peasey A., Pikhart H., Brunner E.J., Bobak M. (2015). Mediterranean diet score and total and cardiovascular mortality in Eastern Europe: The HAPIEE study. Eur. J. Nutr..

[379] Alcalay R.N., Gu Y., Mejia-Santana H., Cote L., Marder K.S., Scarmeas N. (2012). The association between Mediterranean diet adherence and Parkinson’s disease. Mov. Disord..

[380] Otaegui-Arrazola A., Amiano P., Elbusto A., Urdaneta E., Martinez-Lage P. (2014). Diet, cognition, and Alzheimer’s disease: Food for thought. Eur. J. Nutr..

[381] Rodriguez-Morato J., Xicota L., Fito M., Farre M., Dierssen M., de la Torre R. (2015). Potential role of olive oil phenolic compounds in the prevention of neurodegenerative diseases. Molecules.

[382] Singh B., Parsaik A.K., Mielke M.M., Erwin P.J., Knopman D.S., Petersen R.C., Roberts R.O. (2014). Association of mediterranean diet with mild cognitive impairment and Alzheimer’s disease: A systematic review and meta-analysis. J. Alzheimers Dis..

[383] Grosso G., Buscemi S., Galvano F., Mistretta A., Marventano S., La Vela V., Drago F., Gangi S., Basile F., Biondi A. (2013). Mediterranean diet and cancer: Epidemiological evidence and mechanism of selected aspects. BMC Surg..

[384] Schwingshackl L., Hoffmann G. (2014). Adherence to Mediterranean diet and risk of cancer: A systematic review and meta-analysis of observational studies. Int. J. Cancer.

[385] Trichopoulou A., Lagiou P., Kuper H., Trichopoulos D. (2000). Cancer and Mediterranean dietary traditions. Cancer Epidemiol. Biomark. Prev..

[386] Abiemo E.E., Alonso A., Nettleton J.A., Steffen L.M., Bertoni A.G., Jain A., Lutsey P.L. (2013). Relationships of the Mediterranean dietary pattern with insulin resistance and diabetes incidence in the Multi-Ethnic Study of Atherosclerosis (MESA). Br. J. Nutr..

[387] Boghossian N.S., Yeung E.H., Mumford S.L., Zhang C., Gaskins A.J., Wactawski-Wende J., Schisterman E.F. (2013). Adherence to the Mediterranean diet and body fat distribution in reproductive aged women. Eur. J. Clin. Nutr..

[388] Huo R., Du T., Xu Y., Xu W., Chen X., Sun K., Yu X. (2015). Effects of Mediterranean-style diet on glycemic control, weight loss and cardiovascular risk factors among type 2 diabetes individuals: A meta-analysis. Eur. J. Clin. Nutr..

[389] Romaguera D., Norat T., Mouw T., May A.M., Bamia C., Slimani N., Travier N., Besson H., Luan J., Wareham N. (2009). Adherence to the Mediterranean diet is associated with lower abdominal adiposity in European men and women. J. Nutr..

[390] Shai I., Schwarzfuchs D., Henkin Y., Shahar D.R., Witkow S., Greenberg I., Golan R., Fraser D., Bolotin A., Vardi H. (2008). Weight loss with a low-carbohydrate, Mediterranean, or low-fat diet. N. Engl. J. Med..

[391] Sleiman D., Al-Badri M.R., Azar S.T. (2015). Effect of mediterranean diet in diabetes control and cardiovascular risk modification: A systematic review. Front. Public Health.

[392] Vujkovic M., de Vries J.H., Lindemans J., Macklon N.S., van der Spek P.J., Steegers E.A., Steegers-Theunissen R.P. (2010). The preconception Mediterranean dietary pattern in couples undergoing *in vitro* fertilization/intracytoplasmic sperm injection treatment increases the chance of pregnancy. Fertil. Steril..

[393] Schoenaker D.A., Soedamah-Muthu S.S., Callaway L.K., Mishra G.D. (2015). Prepregnancy dietary patterns and risk of developing hypertensive disorders of pregnancy: Results from the Australian Longitudinal Study on Women’s Health. Am. J. Clin. Nutr..

[394] Mikkelsen T.B., Osterdal M.L., Knudsen V.K., Haugen M., Meltzer H.M., Bakketeig L., Olsen S.F. (2008). Association between a Mediterranean-type diet and risk of preterm birth among Danish women: A prospective cohort study. Acta Obstet. Gynecol. Scand..

[395] Karamanos B., Thanopoulou A., Anastasiou E., Assaad-Khalil S., Albache N., Bachaoui M., Slama C.B., El Ghomari H., Jotic A., Lalic N. (2014). Relation of the Mediterranean diet with the incidence of gestational diabetes. Eur. J. Clin. Nutr..

[396] Tobias D.K., Zhang C., Chavarro J., Bowers K., Rich-Edwards J., Rosner B., Mozaffarian D., Hu F.B. (2012). Prepregnancy adherence to dietary patterns and lower risk of gestational diabetes mellitus. Am. J. Clin. Nutr..

[397] Timmermans S., Steegers-Theunissen R.P., Vujkovic M., den Breeijen H., Russcher H., Lindemans J., Mackenbach J., Hofman A., Lesaffre E.E., Jaddoe V.V. (2012). The Mediterranean diet and fetal size parameters: The Generation R Study. Br. J. Nutr..

[398] Vijayakumar R., Walters W.A. (1981). Myometrial prostaglandins during the human menstrual cycle. Am. J. Obstet. Gynecol..

[399] Coyral-Castel S., Rame C., Fatet A., Dupont J. (2010). Effects of unsaturated fatty acids on progesterone secretion and selected protein kinases in goat granulosa cells. Domest. Anim. Endocrinol..

[400] Achache H., Revel A. (2006). Endometrial receptivity markers, the journey to successful embryo implantation. Hum. Reprod. Update.

[401] Sorbera L.A., Asturiano J.F., Carrillo M., Zanuy S. (2001). Effects of polyunsaturated fatty acids and prostaglandins on oocyte maturation in a marine teleost, the European sea bass (*Dicentrarchus labrax*). Biol. Reprod..

[402] Chavarro J.E., Rich-Edwards J.W., Rosner B.A., Willett W.C. (2007). Dietary fatty acid intakes and the risk of ovulatory infertility. Am. J. Clin. Nutr..

[403] Gaskins A.J., Rich-Edwards J.W., Hauser R., Williams P.L., Gillman M.W., Penzias A., Missmer S.A., Chavarro J.E. (2014). Prepregnancy dietary patterns and risk of pregnancy loss. Am. J. Clin. Nutr..

[404] Haugen M., Meltzer H.M., Brantsaeter A.L., Mikkelsen T., Osterdal M.L., Alexander J., Olsen S.F., Bakketeig L. (2008). Mediterranean-type diet and risk of preterm birth among women in the Norwegian Mother and Child Cohort Study (MoBa): A prospective cohort study. Acta Obstet. Gynecol. Scand..

